# Active targeting of orthotopic glioma using biomimetic liposomes co-loaded elemene and cabazitaxel modified by transferritin

**DOI:** 10.1186/s12951-021-01048-3

**Published:** 2021-09-26

**Authors:** Jie Li, Huamin Zeng, Yu You, Rongrong Wang, Tiantian Tan, Weiming Wang, Liyan Yin, Zhaowu Zeng, Yiying Zeng, Tian Xie

**Affiliations:** 1 College of Pharmacy, Chengdu University of Traditional Chinese Medicine, Chengdu, 611137 Sichuan People’s Republic of China; 2 School of Pharmacy, Hangzhou Normal University, Zhejiang 311121 Hangzhou, People’s Republic of China; 3 Key Laboratory of Elemene Class Anti-Cancer Chinese Medicine of Zhejiang Province, Hangzhou, 311121 Zhejiang People’s Republic of China; 4 Engineering Laboratory of Development and Application of Traditional Chinese Medicine from Zhejiang Province, Hangzhou, 311121 Zhejiang People’s Republic of China; 5 Chengdu Ping An Healthcare Medical Examination Laboratory, Chengdu, 611130 Sichuan People’s Republic of China; 6 Traditional Chinese Medicine College of Guangdong Pharmaceutical University, Guangzhou, 511400 People’s Republic of China

**Keywords:** Glioma, Biomimetic liposomes, Homologous-targeting, Immune escaping, Blood–brain barrier, Transferrin

## Abstract

**Background:**

Effective treatment of glioma requires a nanocarrier that can cross the blood–brain barrier (BBB) to target the tumor lesion. In the current study, elemene (ELE) and cabazitaxel (CTX) liposomes were prepared by conjugating liposomes with transferrin (Tf) and embedding the cell membrane proteins of RG2 glioma cells into liposomes (active-targeting biomimetic liposomes, Tf-ELE/CTX@BLIP), which exhibited effective BBB infiltration to target glioma.

**Results:**

The findings showed that Tf-ELE/CTX@BLIP was highly stable. The liposomes exhibited highly significant homologous targeting and immune evasion in vitro and a 5.83-fold intake rate compared with classical liposome (ELE/CTX@LIP). Bioluminescence imaging showed increased drug accumulation in the brain and increased tumor penetration of Tf-ELE/CTX@BLIP in orthotopic glioma model nude mice. Findings from in vivo studies indicated that the antitumor effect of the Tf-ELE/CTX@BLIP led to increased survival time and decreased tumor volume in mice. The average tumor fluorescence intensity after intravenous administration of Tf-ELE/CTX@BLIP was 65.2, 12.5, 22.1, 6.6, 2.6, 1.5 times less compared with that of the control, CTX solution, ELE solution, ELE/CTX@LIP, ELE/CTX@BLIP, Tf-ELE/CTX@LIP groups, respectively. Histopathological analysis showed that Tf-ELE/CTX@BLIP were less toxic compared with administration of the CTX solution.

**Conclusion:**

These findings indicate that the active-targeting biomimetic liposome, Tf-ELE/CTX@BLIP, is a promising nanoplatform for delivery of drugs to gliomas.

**Graphic Abstract:**

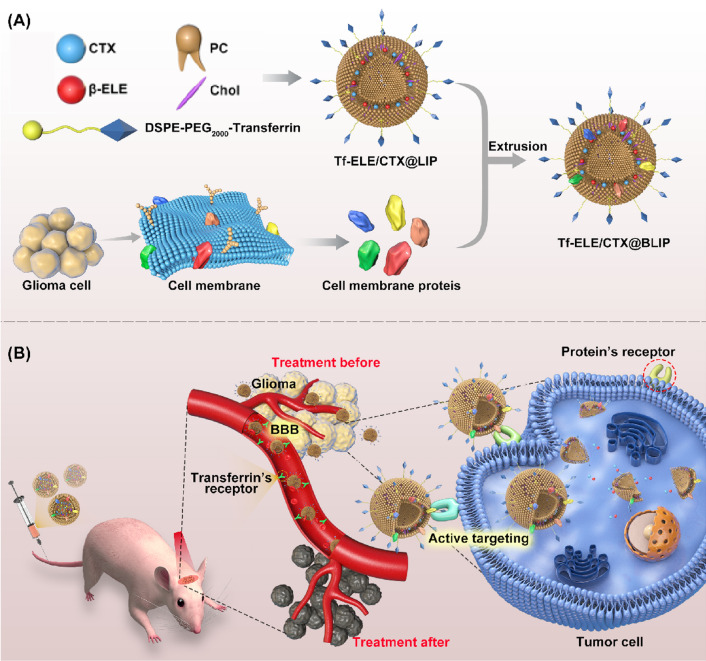

**Supplementary Information:**

The online version contains supplementary material available at 10.1186/s12951-021-01048-3.

## Introduction

Glioma is one of the most threatening malignant brain tumors with a high incidence and mortality rate [1, 2]. Currently, chemotherapy is the first-line and most effective anticancer strategy for glioma owing to its heterogeneity, invasiveness, and inability to be completely resected through surgery [3, 4]. However, chemotherapy is limited by several factors including selective permeability of the blood–brain barrier (BBB), central nervous system toxicity, and low targeting level of drugs at the tumor site [5–9]. In addition, an ATP-dependent efflux pump P-glycoprotein (*P-gp*) occurs on the BBB, which further increases clearance of chemotherapy drugs [10]. Therefore, studies should design a drug delivery vehicle that can penetrate BBB and escape the efflux mechanism to target glioma effectively while exhibiting acceptable neurotoxicity.

Liposomes are cell-like spherical vesicles formed by phospholipids and cholesterol and have recently attracted increased interest owing to their biocompatibility and BBB crossing abilities [11]. However, lack of active tumor targeting property significantly limits their applications in glioma chemotherapy. Several receptors are overexpressed on the BBB, such as insulin, transferrin, endothelial growth factors, and amino acids [12]. Receptor-mediated endocytosis is the main pathway for entry of chemotherapeutic agents to the brain through the BBB [13, 14]. A previous study modified targeting ligand-transferrin (Tf) liposome to specifically recognize transferrin receptors thus increasing BBB penetrating efficiency [15]. Although accumulation of chemotherapeutic agents in the brain was improved through the above approach, the liposomes did not effectively target glioma lesions.

To further improve accumulation of the chemotherapeutic agent at the glioma location, active-targeting biomimetic liposomes were developed by embedding Tf-liposomes with glioma cell membrane proteins (CMP). CMP-based biomimetic nanoengineering has received significant attention recently and applied in studies on biomimetic liposomes [16–18]. The immune system cannot recognize CMP-camouflaged liposomes owing to the specific homologous recognition to the CMP of source cancer cells. Therefore, the biomimetic liposomes exhibit significant targeting and immune escape [19, 20]. CMP-based biomimetic nanoengineering has been widely applied in biomedical fields, such as immunotherapy, bioimaging, theranostics, and glioma phototheranostics since its discovery in 2014 [18, 21–23]. However, to the best of our knowledge, active-targeting biomimetic liposomes of drug co-delivery for glioma chemotherapy have not been fully explored.

Drug combination is an important factor in effective treatment of gliomas [24, 25]. Studies report that cabazitaxel (CTX) inhibits proliferation of cancer cells by binding to tubulin [26]. In addition, CTX is a substrate of *P-gp*, and for *P-gp* has a low affinity for CTX thus it does not easily cause drug resistance [27, 28]. CTX shows significant inhibitory effect on gliomas, however, it is toxic to normal cells [29]. Elemene (ELE) is a fat-soluble small molecular compound which can pass through the BBB and exhibits significant effect on malignant gliomas clinically [30–32]. Previous studies report that ELE and CTX (preferably 5:1 weight ratio) encapsulated in the same nanoparticle exhibit a synergistic effect and shows reduced toxicity [33]. A similar effect was reported when the nanoparticles dosage was only 25% of the conventional CTX injection. Therefore, co-embedding of ELE and CTX in nanoparticles can achieve significant anti-glioma effect.

**Fig. 1:**
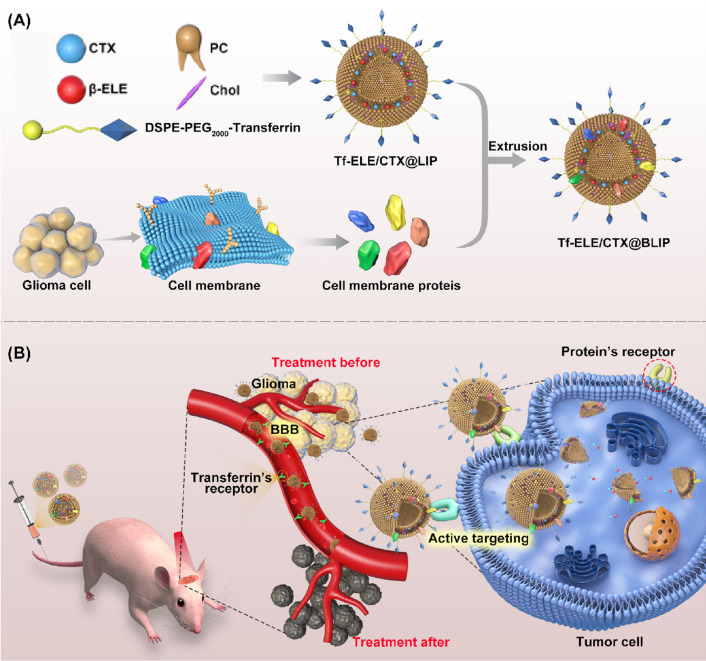
Schematic illustration of the preparation process of active-targeting biomimetic liposomes (Tf-ELE/CTX@BLIP) to optimize BBB penetration and homologous targeted delivery in glioma tumor-bearing mice. **A** Preparation process of Tf-ELE/CTX@BLIP. **B** Schematic diagram showing mechanism of penetration of Tf-ELE/CTX@BLIP through the BBB and delivery of chemotherapy to tumor cells

In the current, ELE and CTX liposomes were constructed by conjugating the two agents with Tf and embedding CMP onto the liposomes (Tf-ELE/CTX@BLIP) to improve transport through the BBB and increase homotypic targeting in glioma-bearing mice (Fig. 1). Activities of Tf-ELE/CTX@BLIP including *P-gp* inhibition, active targeting, immune escape, cytotoxicity assay, and induction of apoptosis in vitro and distribution in vivo were explored. In addition, anti-glioma efficacy and biosafety of Tf-ELE/CTX@BLIP in orthotopic glioma tumor-bearing model was explored. The findings showed that the liposomes improves delivery of the drugs to the glioma site and reduced toxicity of CTX.

## Results

### Preparation and characterization of Tf-ELE/CTX@BLIP

**Fig. 2:**
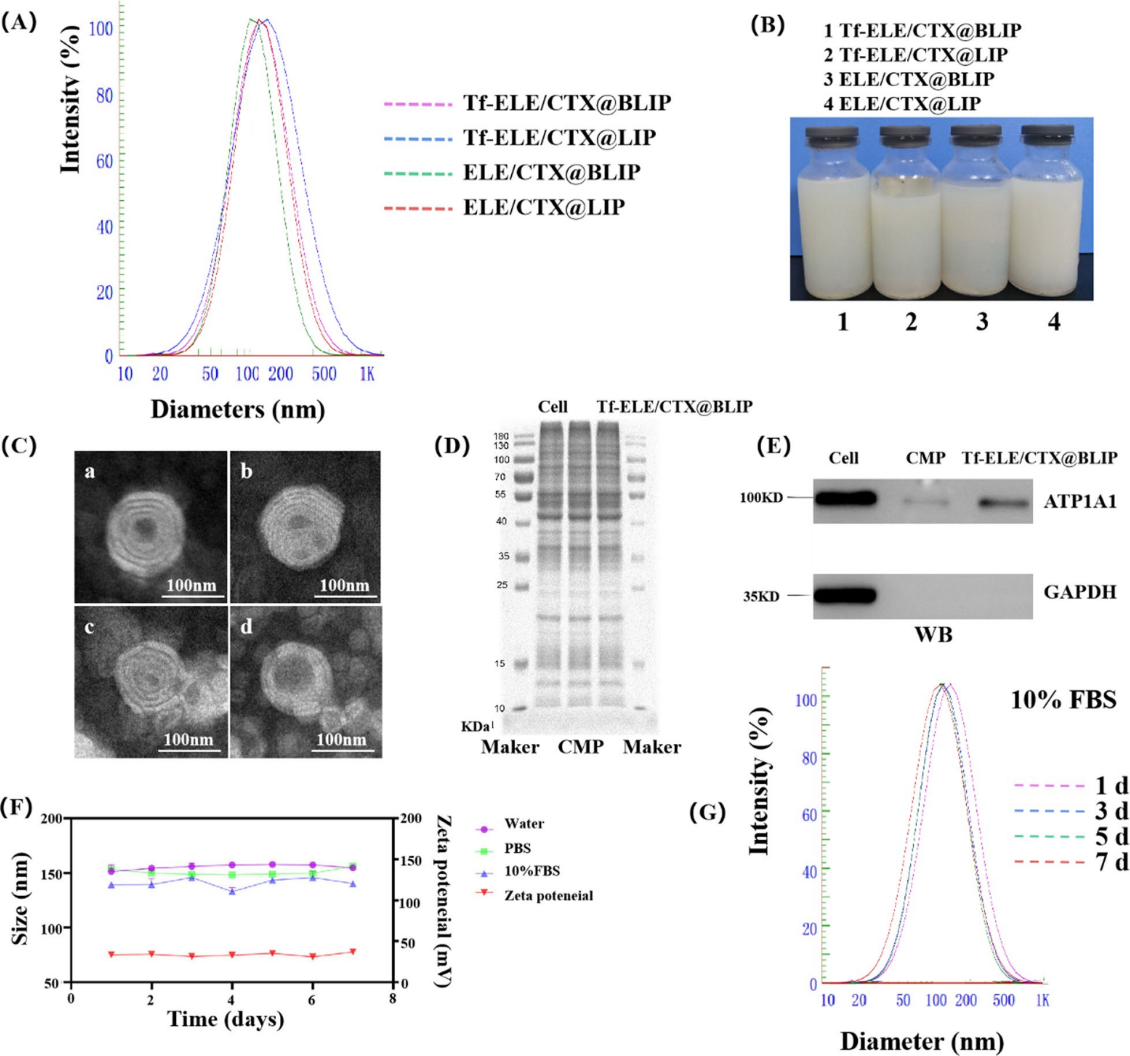
Characterization of biomimetic liposomes and stability evaluation. **A** Particle sizes. **B** Liposome samples. **C** TEM images of Tf-ELE/CTX@BLIP (a), Tf-ELE/CTX@LIP (b), ELE/CTX@BLIP (c) and ELE/CTX@LIP (d). **D**, **E** SDS-PAGE and WB analysis results of Tf-ELE/CTX@BLIP, CMP, and glioma cells. **F** Hydrodynamic diameters and ζ-potential. **G** Stability of TF-ELE /CTX@BLIP diameter in PBS containing 10% FBS after 7-day storage

Analysis showed that the 4 liposome samples were opalescent, translucent liquids and presented different particle sizes (Fig. 2A, B). The mean particle sizes, PDI, ζ-potentials, EE, and DLE of different formulations are listed in Table 1 and Additional file 1: Fig. S1. Particle sizes of liposomes with CMP (Tf-ELE/CTX@BLIP: size 135.1 ± 4.2 nm, PDI = 0.263, and ELE/CTX@BLIP: size 115.7 ± 1.3 nm, PDI = 0.168) were slightly smaller compared with those of liposomes without CMP (Tf-ELE/CTX@LIP size 153.0 ± 3.4 nm, PDI = 0.345, and ELE/CTX@LIP size 135.4 ± 2.0 nm, PDI = 0.205). This finding indicates that CMP embed into the lipid bilayer by continuous extrusion reduced the particle size of traditional liposomes, resulting in a more uniform particle size distribution and smaller PDI, which can further reduce uptake efficiency difference caused by uneven particle size distribution. The findings showed that EE of ELE/CTX was 99.827 ± 0.004% and that of Tf-ELE/CTX@BLIP was 99.106 ± 0.378%. The ζ-potential was 33.57 ± 0.67 mV. Liposomes exhibiting a positive zeta potential are the most widely used type of liposomes. Liposomes with a positive zeta potential are easily polymerized with cell membranes (negative charge) through electrostatic interactions, ensuring complete lysosomal escape through the “proton sponge effect” to prevent lysosomal damage of liposomes. In addition, positive liposomes can easily penetrate the BBB through adsorptive-mediated transcytosis or receptor-mediated transport when triggered by electrostatic interactions when they penetrate the membrane of brain capillary endothelial cells to increase cellular-uptake rate. Therefore, positive liposomes are more suitable for transport across the BBB. TEM images of the four types of liposomes showing their spherical nanostructures are presented in Fig. 2C. The findings showed that ELE/CTX@BLIP and Tf-ELE/CTX@BLIP had an irregular spherical shape for insertion of CMP compared with liposomes without CMP (ELE/CTX@LIP and Tf-ELE/CTX@LIP). SDS-PAGE and WB analysis were performed to explore the effect of ELE/CTX@BLIP and Tf-ELE/CTX@BLIP on protein expression. Analysis showed that the protein profile after treatment of RG2 cells with Tf-ELE/CTX@BLIP was consistent with that of treatment of RG2 cells with CMP (Fig. 2D, E). In addition, WB analysis showed high expression of ATP1A1 (a membrane-specific marker) after treatment with Tf-ELE/CTX@BLIP, indicating that CMPs were successfully embedded onto the surface of Tf-ELE/CTX@BLIP. The findings showed no significant particle size changes after 7 days of storage at 4 ℃, indicating excellent storage stability of Tf-ELE/CTX@BLIP, and a good ζ-potential (Fig. 2F, G). Furthermore, no noticeable size changes in Tf-ELE/CTX@BLIP were observed after storage for 7 days in PBS (pH 7.4) and PBS containing 10% FBS (Additional file 1: Fig. S2), indicating good serum stability of Tf-ELE/CTX@BLIP. This finding indicates its potential application and for in vitro*/*vivo studies.

**Table 1:** Characteristics of the four liposomes (n = 3)

Samples	Size/nm	PDI	ζ-potential/mV	EE (%)		DLE (%)	
				ELE	CTX	ELE	CTX
Tf-ELE/CTX@BLIP	135.1 ± 4.2	0.263 ± 0.018	33.57 ± 0.67	99.827 ± 0.004	99.106 ± 0.378	16.376 ± 0.001	1.640 ± 0.006
Tf-ELE/CTX@LIP	153.0 ± 3.4	0.345 ± 0.011	54.96 ± 1.91	99.296 ± 0.397	99.419 ± 0.104	16.113 ± 0.064	1.609 ± 0.002
ELE/CTX@BLIP	115.7 ± 1.3	0.168 ± 0.023	33.95 ± 1.08	99.767 ± 0.001	97.175 ± 0.013	15.963 ± 0.001	1.555 ± 0.0002
ELE/CTX@LIP	135.4 ± 2.0	0.205 ± 0.021	34.53 ± 2.66	97.701 ± 0.397	95.640 ± 0.218	15.826 ± 0.064	1.545 ± 0.004
CMP	–	–	30.12 ± 0.73	–	–	–	–

### Homologous targeting and immune escape abilities of Tf-ELE/CTX@BLIP

**Fig. 3:**
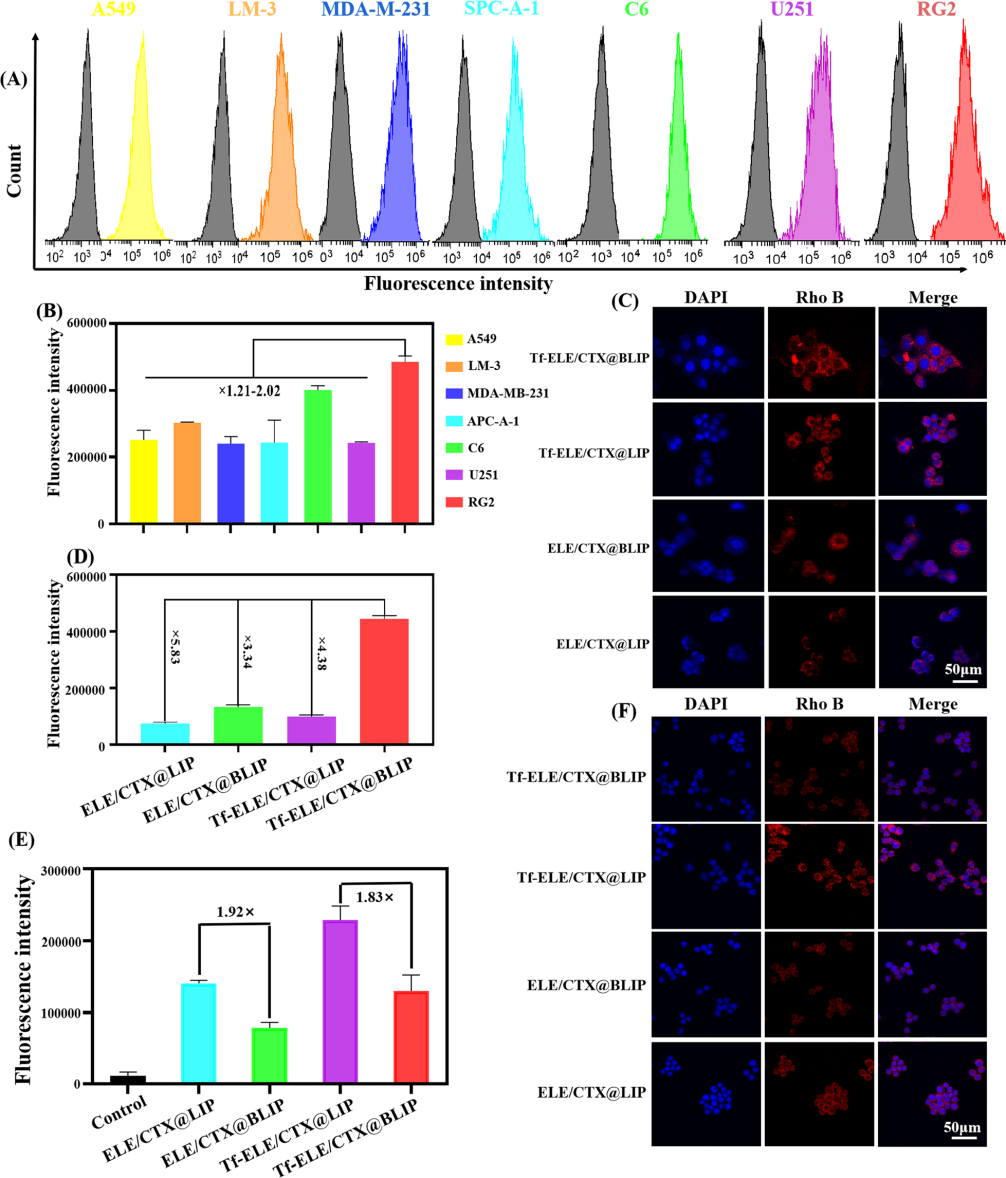
In vitro evaluation of immune escape and homologous targeting characteristics of Tf-ELE/CTX@BLIP. **A**, **B** Flow cytometry analysis and fluorescent quantification of A549, LM-3, SPC-A-1, MDA-M-231, U251, C6, and RG2 cells treated with Tf-ELE/CTX@BLIP for 2 h. **C**, **D** CLSM images and fluorescent quantification of RG2 glioma cells treated with Tf-ELE/CTX@BLIP, Tf-ELE/CTX@LIP, ELE/CTX@BLIP, and ELE/CTX@LIP for 2 h. **E**, **F** Fluorescent quantification and CLSM images of RAW264.7 cells after incubation with Tf-ELE/CTX@BLIP, Tf-ELE/CTX@LIP, ELE/CTX@BLIP, and ELE/CTX@LIP for 2 h. Rho B = 20 μg/mL. Scale bar = 50 μm

Cellular uptake of Tf-ELE/CTX@BLIP by several different cell lines including A549, LM-3, SPC-A-1, MDA-M-231, U251, C6, and RG2 was evaluated to explore the specific homologous targeting abilities of Tf-ELE/CTX@BLIP in cancer cells. The findings showed that the fluorescence intensity of Tf-ELE/CTX@BLIP in the RG2 cell group was 1.21- to 2.02-fold higher compared with that of other cell lines (Fig. 3A, B). This finding indicates that Tf-ELE/CTX@BLIP exhibited higher uptake efficiency in RG2 cells and further implied that the homologous adhesion property was successfully transferred to the liposomes.

Uptake of Tf-ELE/CTX@BLIP by RG2 cells was further evaluated by CLSM. A higher amount of Tf-ELE/CTX@BLIP was ingested into the cytoplasm of the RG2 cell, after incubation at 37 ℃ for 2 h compared with the amount of ELE/CTX@LIP, Tf-ELE/CTX@LIP, ELE/CTX@BLIP, and Tf-ELE/CTX@BLIP (Fig. 3C). This indicates that Tf-ELE/CTX@BLIP acquired a high homologous targeting ability. Quantitative analysis by flow cytometry showed that the fluorescence intensity for Tf-ELE/CTX@BLIP-treated cells was 3.34- to 5.83-fold stronger compared with that of other groups (Fig. 3D, Additional file 1: Fig. S3), indicating its excellent active targeting ability. The uptake efficiency of TF-ELE /CTX@BLIP in RG2 was significantly higher compared with that of C6 and U251 cells (Additional file 1: Fig. S4).

Moreover, internalization of Tf-ELE/CTX@BLIP, Tf-ELE/CTX@LIP, ELE/CTX@BLIP, and ELE/CTX@LIP by RAW264.7 macrophages was explored by flow cytometry and CLSM imaging to determine changes in their antiphagocytic properties. The findings showed that only a few Tf-ELE/CTX@BLIP and ELE/CTX@BLIP were internalized by RAW 264.7 macrophages (Fig. 3E, F Additional file 1: Fig. S5). In addition, treatment of cells with Tf-ELE/CTX@BLIP and ELE/CTX@BLIP exhibited 1.83- and 1.92-fold weaker fluorescence intensity compared with that of Tf-ELE/CTX@LIP and ELE/CTX@LIP treated cells, indicating the excellent immune escape characteristics of biomimetic liposomes. Notably, rate of phagocytosis of RAW 264.7 macrophages was enhanced after addition of Tf.

### In vitro cytotoxicity assay and cell apoptosis of Tf-ELE/CTX@BLIP

**Fig. 4:**
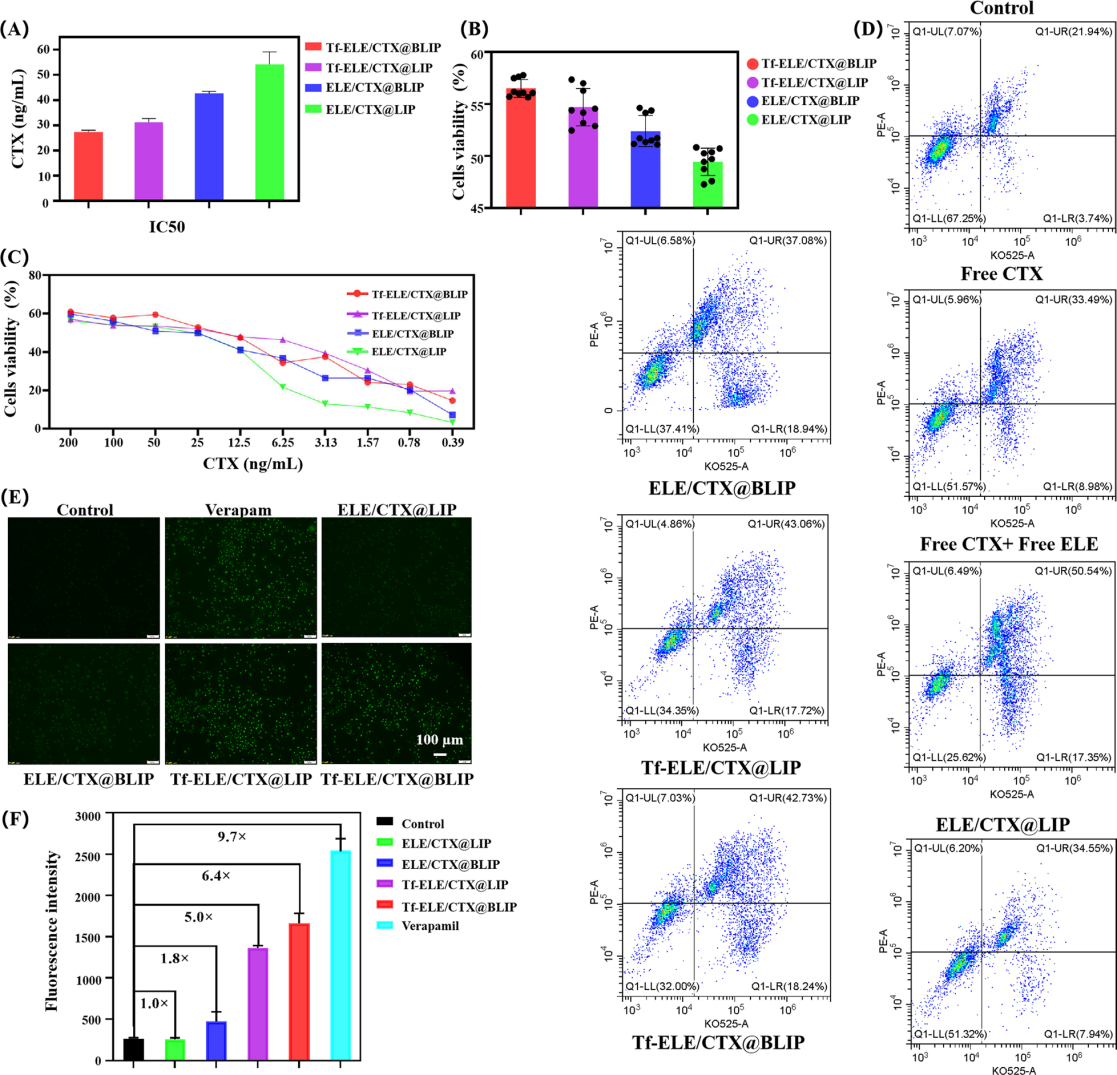
In vitro cytotoxicity assay and apoptosis of Tf-ELE/CTX@BLIP. **A** IC50, **B** Cell viability (%) (C_CTX_ = 50 ng/mL) and **C** Cell viability (C_CTX_ 0.4 to 200 ng/mL) of RG2 cells treated with ELE/CTX@LIP, ELE/CTX@BLIP, Tf-ELE/CTX@LIP and Tf-ELE/CTX@BLIP for 48 h. **D** Representative results of staining and quantitative analysis of RG2 glioma cells after incubation with Tf-ELE/CTX@BLIP, Tf-ELE/CTX@LIP, ELE/CTX@BLIP, and ELE/CTX@LIP for 48 h. **E** Rho 123 uptake and **F** Quantitative analysis of Rho 123 uptake by bEnd.3 cells by flow cytometry after incubation with Tf-ELE/CTX@BLIP, Tf-ELE/CTX@LIP, ELE/CTX@BLIP, and ELE/CTX@LIP for 2 h. Rho 123 = 20 μg/mL. Scale bar = 100 μm

Findings from CCK-8 quantitative analysis indicated that the IC50s of ELE/CTX@LIP, ELE/CTX@BLIP, Tf-ELE/CTX@LIP, and Tf-ELE/CTX@BLIP (C_CTX_ 0.4 to 200 ng/mL) groups were 27.38 ± 0.67, 31.30 ± 1.44, 42.70 ± 0.76, and 54.25 ± 4.90 ng/mL, respectively (Fig. 4A). The findings indicate a significantly higher effect of Tf-ELE/CTX@BLIP against glioma cells compared with other liposomes. The cell inhibition ratio for Tf-ELE/CTX@BLIP, Tf-ELE/CTX@LIP, ELE/CTX@BLIP and ELE/CTX@LIP (C_CTX_ = 50 ng/mL) was 56.50 ± 0.86%, 54.69 ± 1.79%, 52.41 ± 1.50% and 49.44 ± 1.33%, respectively (Fig. 4B, C). The higher cytotoxicity induced by Tf-ELE/CTX@BLIP in RG2 cells indicates potential for further in vivo research and applications.

A dual staining assay was performed using flow cytometry to analyze apoptotic cells quantitatively. A significant increase in number of apoptotic cells was observed in RG2 cells post-incubation with Tf-ELE/CTX@BLIP (60.97%), ELE/CTX@BLIP (56.02%), Tf-ELE/CTX@LIP (60.80%), and ELE/CTX@LIP (42.49%), compared with the control group (25.68%), CTX solution group (42.47%) and ELE + CTX solution group (67.89%) (Fig. 4D). These findings show that ELE + CTX inhibits proliferation of glioma cells by inducing the apoptosis pathway. In addition, Tf-ELE/CTX@BLIP promotes apoptosis through conjugation of Tf and CMP present in the liposomes, which is consistent with findings from the cytotoxicity assay.

### Inhibitory effect of Tf-liposomes on *P-gp* function

Transferrin conjugated liposomes can be selectively ingested by the brain capillary endothelial cells (through transferrin receptor-mediated endocytosis) to enhance brain absorption of drugs. Ferritin receptor-mediated endocytosis is one of the main ways for delivery drugs to the brain. *P-gp* located on the lumen surface of endothelial capillaries plays a key role in drug uptake and efflux. Dysregulation of *P-gp* is associated with main physiological disorders of the BBB (the efflux effect). Treatment with verapamil (a *P-gp* efflux inhibitor) as a positive control, the fluorescent substrate, Rho 123 of *P-gp*, can be used to explore the efflux effect of the liposomes. Accumulation of Rho 123 in bEnd.3 cell incubated with Tf-ELE/CTX@BLIP, Tf-ELE/CTX@LIP, ELE/CTX@BLIP, and ELE/CTX@LIP was determined to explore delivery of different formulations across the BBB. The findings showed that the efflux of Rho 123 from bEnd.3 cells after incubation with Tf-ELE/CTX@LIP and Tf-ELE/CTX@BLIP were significantly reduced compared with the control group, implying that Tf-ELE/CTX@BLIP and Tf-ELE/CTX@LIP significantly improved movement of Rho 123 through the BBB (Fig. 4E). Flow cytometry analysis showed that the fluorescence intensity in Tf-ELE/CTX@BLIP and Tf-ELE/CTX@LIP-treated cells was significantly increased (Fig. 4F), indicating a suppressed efflux effect. Moreover, relative expression of *P-gp* on bEnd.3 cells after incubation with the Tf-ELE/CTX@BLIP and Tf-ELE/CTX@LIP was decreased compared with that of the control and unmodified liposomes (ELE/CTX@BLIP and ELE/CTX@LIP) (Additional file 1: Fig. S6). This findings indicates that liposomes modified with Tf inhibited *P-gp* efflux of chemotherapeutic drugs.

### In vivo homologous targeting bioluminescence imaging

**Fig. 5:**
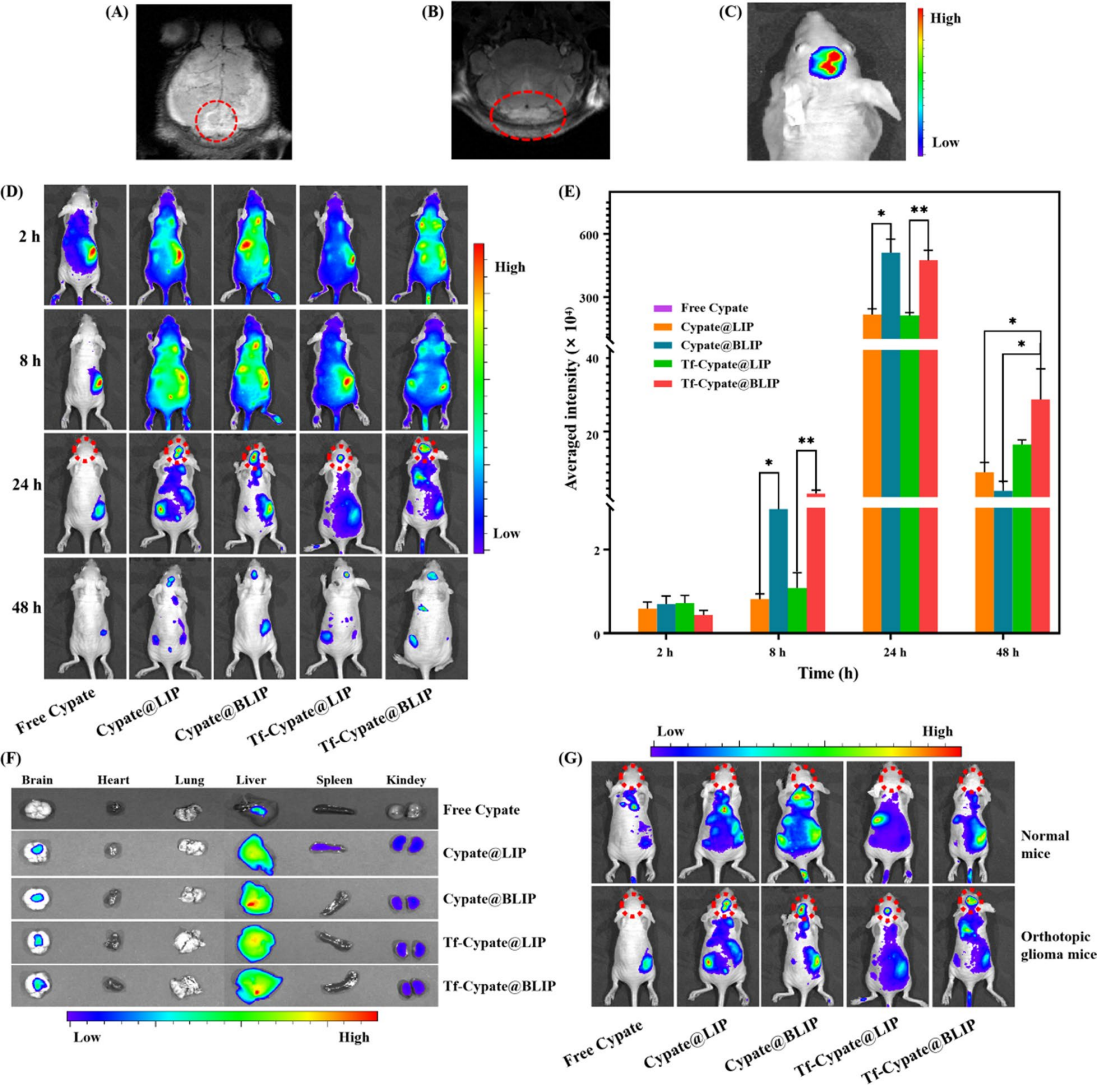
In vivo bioluminescence imaging after administration of Tf-Cypate@BLIP, Tf-Cypate@LIP, Cypate@BLIP, and Cypate@LIP in nude mice bearing glioma 15 days post glioma transplantation. **A**, **B** Coronal and transverse cutaway view by MRI. **C** In vivo fluorescence images. **D** Bioluminescence signal distribution. **E** Bioluminescence quantitative analysis of glioma region at different time points. **F** Ex vivo bioluminescence images of tissues from the brain, liver, heart, spleen, lung, and kidney at 48 h post-injection. **G** Bioluminescence signal distribution in normal and orthotopic glioma models. C_Cypate_ = 0.5 mg/kg. *p < 0.05, **p < 0.01

Glioma cells were implanted into the striatum of mice and an orthotopic glioma model was established to further explore the active targeting ability of liposomes modified with CMP/Tf and to determine the distribution in vivo. Fluorescence imaging was performed to determine the luciferase activity of glioma cells. MRI was performed for analysis of glioma lesion in nude mice 15 days post glioma cell implantation (Fig. 5A, B). Glioma cells showed excellent proliferation activity, as shown by the high fluorescence signal of the tumor (Fig. 5C). In addition, Tf-Cypate@BLIP, Tf-Cypate@LIP, Cypate@BLIP, Cypate@LIP, and free Cypate were administered into normal mice, and the orthotopic glioma model through the tail vein. Analysis did not show bioluminescence signal in brains of mice treated with free Cypate (Fig. 5D, E). The average intensity of Tf-Cypate@BLIP, Tf-Cypate@LIP, Cypate@BLIP, Cypate@LIP group were significantly different, indicating that the enhanced permeability and retention effect dominated the early stage of tumor growth. However, Tf-Cypate@BLIP and Cypate@BLIP with specific homologous targeting and immune evasion abilities easily crossed the BBB and specifically recognized homologous cells. Moreover, Tf can inhibited the efflux of *P-gp*, allowing more drugs to accumulate in the brain for a longer period (Fig. 5D). Analysis of ex vivo bioluminescence images showed that the liposomes were mainly distributed in the glioma region and liver and slightly distributed in the kidney (Fig. 5F, Additional file 1: Fig. S7), indicating high tumor targeting and potential hepatorenal metabolic pathways of liposomes, which was consistent with the quantitative analysis results. Furthermore, the high fluorescent signal indicates that Tf-ELE/CTX@BLIP had high infiltrating ability through the damaged BBB and arriving at the glioma region compared with other liposomes (Additional file 1: Fig. S7). However, accumulation of liposomes in the liver and kidney may cause hepatorenal toxicity, therefore, further experiments are should be conducted to explore the safety of the liposomes. Moreover, high fluorescent signal was observed in the glioma-bearing mice brain. On the contrary, only a few liposomes were absorbed by brain tissue of normal mice, and the findings showed that they were enriched for a shorter time (Fig. 5G, Additional file 1: Fig. S8). This finding indicated that glioma cells entered the brain and grew rapidly, causing damage to the BBB. Notably, liposomes did not enter normal brain tissue, which was consistent with the observation of significantly higher expression of Tf receptor in capillary endothelial cells of tumor-bearing mice brain compared with that observed in normal mice brain tissue.

### In vivo anti-tumor efficacy of Tf-ELE/CTX@BLIP

**Fig. 6:**
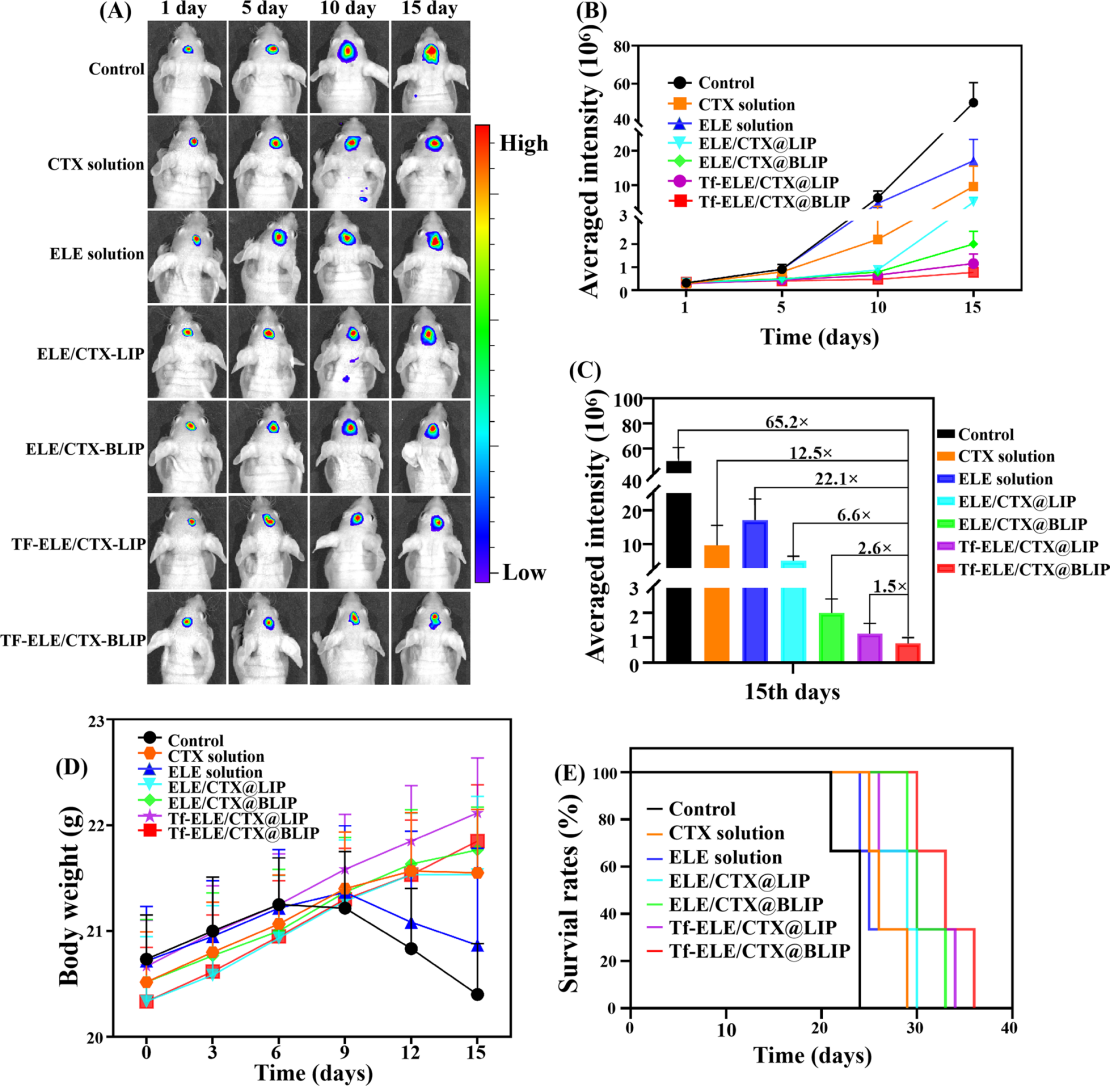
In vivo antitumor efficacy of Tf-ELE/CTX@BLIP in nude mice bearing orthotopic glioma. **A** Representative fluorescent images for different treatment groups. **B**, **C** Average fluorescent intensity of brain tissues from the different groups. **D**, **E** Bodyweight and survival rate curves for mice in the different treatment groups

Fluorescence imaging was performed to explore anti-glioma effect of the liposomes in vivo. The findings showed that the fluorescence signal in brain tumors of glioma-bearing mice treated with Tf-ELE/CTX@BLIP was significantly lower compared with that of other groups after 15 days of treatment (Fig. 6A). Quantitative analysis and tumor fluorescent intensity showed a tumor growth suppressing effect by Tf-ELE/CTX@BLIP, as indicated by an average fluorescence intensity of 65.2, 12.5, 22.1, 6.6, 2.6, 1.5 times weaker compared with that of the control, CTX solution, ELE solution, ELE/CTX@LIP, ELE/CTX@BLIP, Tf-ELE/CTX@LIP groups, respectively. (Fig. 6B, C, Additional file 1: Fig. S9). In addition, ELE/CTX@BLIP, Tf-ELE/CTX@LIP, and Tf-ELE/CTX@BLIP groups exhibited a significant therapeutic effect on in-situ glioma in nude mice. CTX solution and ELE/CTX@LIP inhibited growth of in-situ glioma through the effect was not significant. Tf-ELE/CTX@BLIP group showed the lowest tumor fluorescence signal compared with other groups after 15-day administration. These findings indicate that Tf-ELE/CTX@BLIP-mediated chemotherapy can significantly inhibit glioma growth. Moreover, median survival time (MST) and body weight showed that Tf-ELE/CTX@BLIP exhibited anti-glioma effect (Table 2, Fig. 6D, E). Body weights of the control, ELE solution groups significantly decreased from day 9 of treatment; however, of the body weight of mice treated with CTX solution, ELE/CTX@LIP, ELE/CTX@BLIP, Tf-ELE/CTX@LIP and Tf-ELE/CTX@BLIP groups showed an increase during the 15 days after administration. This finding implies that drug-loaded liposomes exhibited beneficial antitumor efficacy to improve the physical condition of mice. MST of the ELE/CTX@LIP group was 28 days, indicating better anti-glioma effect compared with the control, CTX solution, and ELE solution groups. The MST of ELE/CTX@BLIP and Tf-ELE/CTX@LIP treated groups was 31 and 30 days, respectively, indicating that MST was improved compared with that of groups administered with CTX solution and traditional liposome. The finding indicated that targeting liposomes exhibited higher antiglioma effect. The MST of the Tf-ELE/CTX@BLIP group was 33 days, high by 6.5% and 10.0% compared with that of the ELE/CTX@BLIP and Tf-ELE/CTX@LIP groups, indicating that Tf and CMP synergistically contributed to the anti-glioma effect of Tf-ELE/CTX@BLIP, which can be attributed to brain accumulation of the drugs and homologous gliomas targeting.

**Table 2:** Survival time of nude mice bearing orthotopic glioma after treatment (n = 3)

Groups	Median (days)	Mean survival (days)	Maximal survival (days)	Incremental survival time (%)	*p* value (vs control)	*p* value (vs CTX solution)
Control	23	23.0 ± 1.7	24	**–**	**–**	0.0295
CTX solution	27	26.7 ± 2.1	29	15.94	0.0295	**–**
ELE solution	26	26.0 ± 2.6	29	13.04	0.096	0.1615
ELE/CTX@LIP	28	28.0 ± 2.6	30	21.74	0.0295	0.3722
ELE/CTX@BLIP	31	30.7 ± 2.1	33	33.33	0.0295	0.0629
Tf-ELE/CTX@LIP	30	30.0 ± 4.0	34	30.43	0.0295	0.1341
Tf-ELE/CTX@BLIP	33	33.0 ± 3.0	36	43.48	0.0295	0.0246

**Fig. 7:**
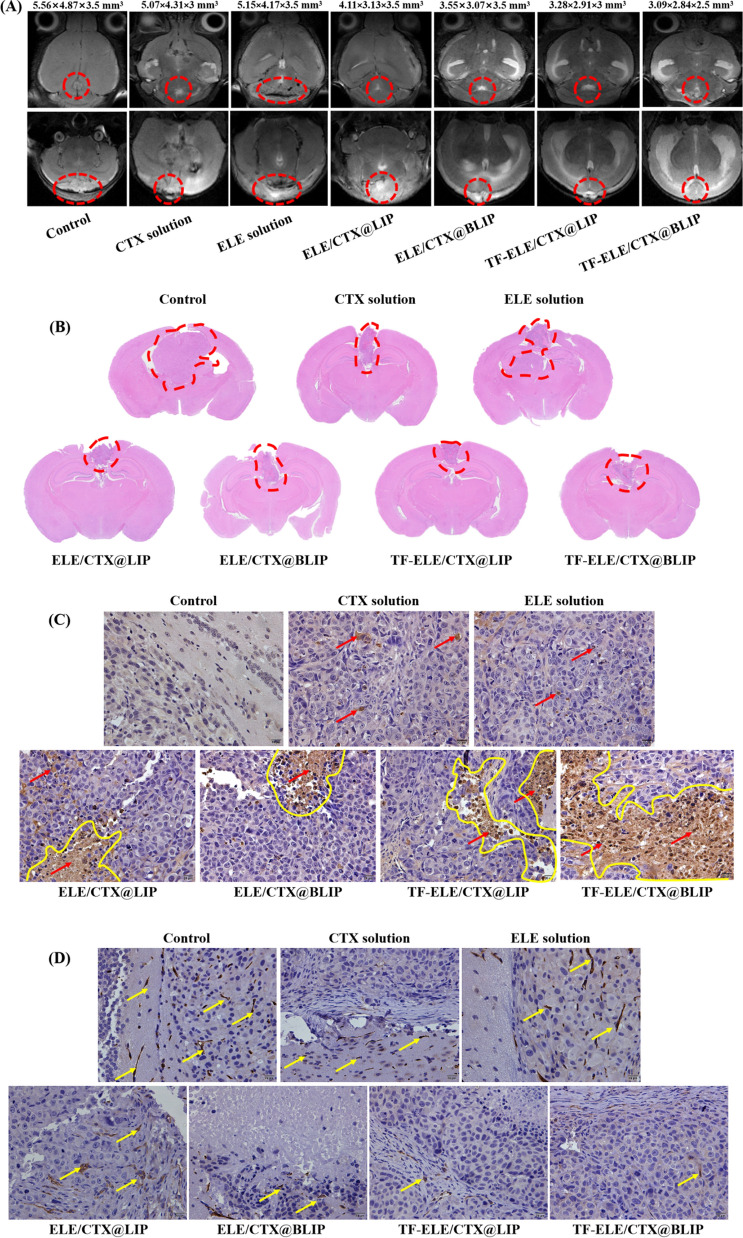
Anti-tumor effect of Tf-ELE/CTX@BLIP on brain of nude mice bearing orthotopic glioma. **A** MRI analysis of glioma in different groups. The first and second row represents transverse and coronal section view, respectively. **B** H&E staining, × 40. **C** TUNEL analysis. Arrow indicates apoptotic cells, × 200. **D** *P-gp* expression level in nude mice bearing orthotopic glioma after treatment. Arrow indicates positive expression, × 200

MRI is used for direct observation of brain areas. In the current study, MRI was used to explore tumor progression after 15 days of cell inoculation. The findings showed less tumor region in the Tf-ELE/CTX@BLIP-treated mice compared with other groups (Fig. 7A). In addition, Tf-ELE/CTX@BLIP exhibited higher glioma suppressive effect after 15 days implantation of glioma cells compared with other groups. Irregular isometric T1 mixed T2 space-occupying lesions which were surrounded by patchiness and macular edema zones were observed in the brains of glioma-bearing mice in the control group and the group administered with ELE solution. The adjacent brain tissue was oppressed by the tumor, showing glial hyperplasia and unclear boundaries. The left lateral ventricle was narrowed and the midline shifted to the left. The brain lesions in CTX solution group mice were similar to those observed in the control group and ELE solution group, however, no glial hyperplasia was observed. Mice in the ELE/CTX@LIP group showed the same type of lesion, accompanied by edema around the lesion compared with mice in the control and ELE solution groups. Compression of the tumor in the adjacent brain tissue narrowed the left lateral ventricle and shifted the midline to the left. Notably, no edema was observed around the brain lesions for mice in ELE/CTX@BLIP group and Tf-ELE/CTX@LIP group, and the adjacent brain tissue was not oppressed, and it showed a distinct middle line. Mice in the Tf-ELE/CTX@BLIP group showed a clearly visible boundary and midline, with no edema around the lesion and no compression of the tumor in the adjacent brain tissue. This finding indicated the invasive growth of tumor cells was significantly inhibited by Tf-ELE/CTX@BLIP.

To explore the potential antitumor mechanism, brain tissue slices of nude mice bearing orthotopic glioma were analyzed. H&E staining of brain tissue slices from the Tf-ELE/CTX@BLIP treated group showed that the texture of tumor tissue was tight, did not exhibit evident apoptotic cells and there was no central necrotic area. The necrotic area was less than 1/4 of the total area, which was consistent with the findings that this group exhibited shorter MST compared with other groups (Fig. 7B, Additional file 1: Fig. S10). Tumor size of the free ELE and CTX solution groups mice was reduced, with a small (ELE solution group) or higher (CTX solution group) number of apoptotic cells, indicating a definite anti-glioma effect of ELE and CTX. The findings showed that glioma size of mice in the ELE/CTX@LIP, ELE/CTX@BLIP and Tf-ELE/CTX@LIP groups were significantly reduced compared with glioma size in mice treated with ELE & CTX. The texture of glioma mice in ELE/CTX@LIP, ELE/CTX@BLIP and Tf-ELE/CTX@LIP groups was loose, with higher number of apoptotic cells and a central necrotic area. The necrotic area was approximately half of the total area, indicating a significant anti-glioma effect. Notably, the Tf-ELE/CTX@BLIP group showed the smallest glioma size, and the necrosis area was approximately 3/4 of the total tumor area, indicating that the active-targeting biomimetic nanoplatform exhibited the highest anti-glioma effect compared with the other treatments.

Brain tissue slices were stained and analyzed by TUNEL immunofluorescence to explore the apoptosis-inducing ability of the liposomes. Highest apoptosis rate was observed for the Tf-ELE/CTX@BLIP-treated mice compared with that of mice in the other groups, which was consistent with results from cell apoptosis analysis (Fig. 4D). This finding indicated that ELE/CTX@LIP, ELE/CTX@BLIP, Tf-ELE/CTX@LIP and Tf-ELE/CTX@BLIP promoted tumor apoptosis in vivo*,* whereas apoptotic activity of the control group was negligible (Fig. 7C). Furthermore, *P-gp* expression level was evaluated. The findings showed that Tf-ELE/CTX@BLIP group had the lowest *P-gp* expression compared with the control group, ELE/CTX solution group and classical liposome group, implying that Tf-ELE/CTX@BLIP did not upregulate *P-gp* expression but it exhibited efflux effect (Fig. 7D).

### In vivo biosafety evaluation of Tf-ELE/CTX@BLIP

**Fig. 8:**
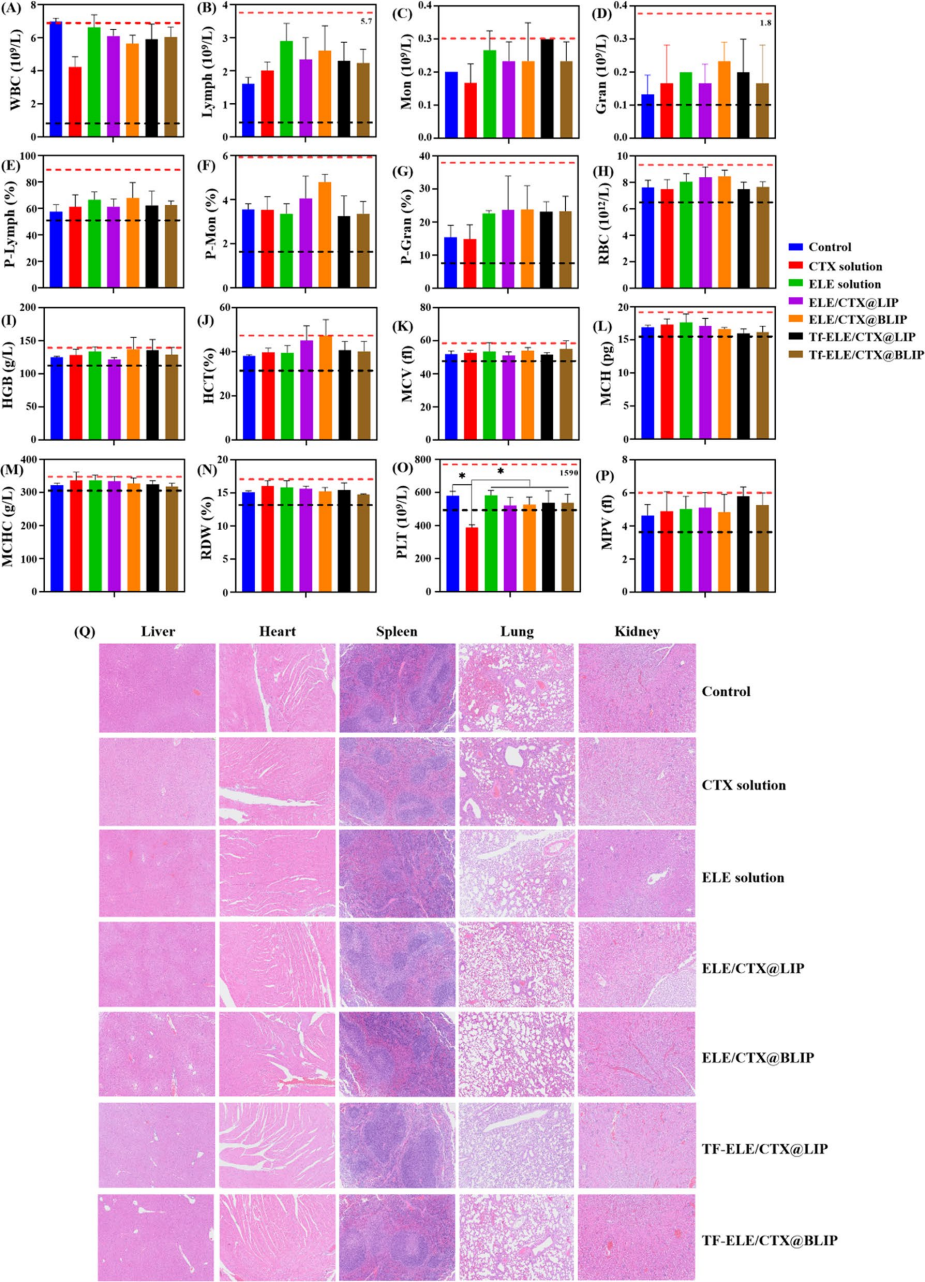
In vivo biosafety of Tf-ELE/CTX@BLIP. **A**–**P** Blood routine examination after treatment with control, CTX solution, ELE solution, ELE/CTX@LIP, ELE/CTX@BLIP, Tf-ELE/CTX@LIP and Tf-ELE/CTX@BLIP. **A** WBC: white blood cell. **B** Lymph: lymphocyte. **C** Mon: monocyte. **D** Gran: neutrophil granulocyte. **E** P-Lymph: percentage of lymphocyte. **F** P-Mon: percentage of monocyte. **G** P-Gran: percentage of neutrophil granulocyte. **H** RBC: red blood cell. **I** HGB: hemoglobin. **J** HCT: hematocrit. **K** MCV: mean corpuscular volume. **L** MCH: mean corpuscular hemoglobin. **M** MCHC: mean concentration of corpuscular hemoglobin. **N** RDW: red blood cell distribution width. **O** PLT: platelet. **P** MPV: mean platelet volume. The region between black and red lines represents the normal parameter range. *p < 0.05, NS indicates no significance. **Q** H&E staining of the major organs harvested from mice sacrificed on day 15 after treatment

Several blood routine parameters were evaluated to explore the biosafety of Tf-ELE/CTX@BLIP. Tf-ELE/CTX@BLIP, Tf-ELE/CTX@LIP, ELE/CTX@BLIP, ELE/CTX@LIP, ELE solution, CTX solution or physiological saline were administered to nude mice bearing orthotopic glioma through the tail vein. The findings shoed that most of the blood routine parameters of the experimental groups were within the normal physiological reference range compared with the control group (Fig. 8A–P), implying that the experimental groups did not exhibit hematological toxicity in vivo, except for the platelet count after administration of CTX solution. In addition, total bilirubin, blood urea nitrogen, uric acid, ALT, creatinine and AST levels of mice administered with Tf-ELE/CTX@BLIP, Tf-ELE/CTX@LIP, ELE/CTX@BLIP, ELE/CTX@LIP, ELE solution were similar with the levels of the control group, indicating that the liposomes did not exhibit significant hepatorenal toxicity in mice (Additional file 1: Fig. S11). The findings showed significant tubular lesion in kidneys of the CTX solution treated group compared with the control group, indicating that CTX exerted nephrotoxic effects (Fig. 8Q). H&E staining results of liver, heart, spleen, lung and kidney in other groups did not show significant or showed only minor abnormal and inflammatory cell infiltration compared with the control group. This finding indicating that the encapsulation of the liposome reduced nephrotoxicity effect of CTX. These findings indicate that Tf-ELE/CTX@BLIP has good biocompatibility for treatment of glioma.

## Discussion

In the current study, Tf and glioma CMP were used to modify classical liposomes to form active-targeting biomimetic, Tf-ELE/CTX@BLIP, for treatment of orthotopic glioma. Tf-ELE/CTX@BLIP were prepared by conjugating liposomes with transferrin (Tf) and embedding the cell membrane proteins of RG2 glioma into liposomes, which exhibited high efficacy in infiltration of the BBB and targeting of glioma cells. Tf-ELE/CTX@BLIP is a highly stable drug carrier with significant homologous targeting and is capable of immune evasion. Tf-ELE/CTX@BLIP enhanced accumulation of drugs in the brain and increased tumor penetration resulting to increased survival time and decreased tumor volume owing to the presence of Tf and CMP.

Traditional liposome is a type of vesicle with cell-like structure, which can be loaded with fat-soluble and water-soluble drugs and improve their bioavailability. In addition, liposome entrapment can increase the tumor uptake rate of drugs in vivo. However, traditional liposomes are not effective in treatment of tumors without modification, mainly for brain tumors. Studies report presence of over-expressed transferrin receptors in brain capillary endothelial cells, which can selectively coordinate Tf [34, 35]. Therefore, using Tf modified liposomes can improve drug transport across the BBB and enhance brain absorption through receptor-mediated endocytosis. Therefore, modification of liposomes by addition of Tf is effective approach for development of drugs targeted to the brain. Moreover, some researchers have proposed use of CMP as tumor selective targets to modify liposomes thus improving tumor targeted drug delivery owing to the specific and homologous expression of tumor membrane proteins in human malignant tumors. However, active-targeting biomimetic liposomes modified by Tf and CMP have not been reported previously.

Previous studies prepared a variety of Tf-modified nano-preparations, including liposomes, and reported that the transport efficiency of modified drug-containing liposomes across the BBB was significantly higher compared with that of free drugs, indicating that Tf-liposomes can effectively transport drugs across the brain [36, 37]. In the current study, four types of liposomes including Tf-ELE/CTX@BLIP, Tf-ELE/CTX@LIP, ELE/CTX@BLIP and ELE/CTX@LIP were prepared. The findings showed that the entrapment efficiency of the liposomes was more than 97%, and the entrapment efficiency of the drug was significantly increased by of loading Tf or CMP through the extrusion process. The therapeutic effects of Tf-ELE/CTX@BLIP with unmodified or single modified liposomes on glioma cells in vitro and anti-glioma effects in vivo were explored. In vivo and in vitro findings showed that Tf-ELE/CTX@BLIP was the most effective nano-platform for drug transport. However, the mechanism of entry of Tf to the brain should be explored further. In the current study, an activity-targeted bionic liposome modified with both Tf and CMP was constructed to improve transport and uptake efficiency of CTX/ELE into brain/glioma. *P-gp* is located on the lumen surface of endothelial capillaries and is a ABC transporter that plays a key role in drug uptake and outflow. In addition, it is one of the major physiological barriers on the BBB. ELE/CTX are fat-soluble drugs, and BBB significantly limits their brain absorption, thus their bioavailability is significantly low. The penetration ability of the liposome through the BBB was explored through in vitro (accumulation of Rho 123 in bEnd.3 cells was determined) and in vivo studies (through bioluminescence imaging of liposomes in nude mice bearing orthotopic glioma after 15 days glioma transplantation). The findings showed that expression levels of *P-gp* and efflux of Rho123 in bEnd.3 cells incubated with Tf-ELE/CTX@LIP and Tf-ELE/CTX@BLIP liposomes decreased significantly compared with the levels of the control group, ELE/CTX solution group and classical liposome group, indicating that Tf modified liposomes significantly improved effectiveness of drug delivery through BBB by bypassing *P-gp* or inhibiting expression of *P-gp*.

Notably, ELE/CTX combined with liposomes improved the therapeutic effect of CTX and reduced toxicity of CTX. The findings showed that the platelet index of mice in the CTX solution group was significantly lower compared with that of mice in the control group, indicating that treatment with CTX solution exhibited hematologic toxicity. In addition, the kidneys of mice in the CTX solution group showed significant typical renal tubular lesions, indicating that CTX induced nephrotoxicity. Notably, these results were consistent with findings from previous studies on toxicity of CTX [38]. However, there were no evident abnormalities or only slight inflammatory cell infiltration was observed in kidneys and lungs of the other groups. This finding indicates that drug combination with liposome reduces the renal toxicity of CTX. The possible mechanisms of synergistic effect and reduced toxicity were summarized as follows: (1) Tumor cell inhibitory effect of ELE. However, ELE shows obvious anti-cancer activity only with a larger dosage, because the faster clearance efficiency than CTX in the body [33]. Therefore, the anti-cancer effect of ELE is more likely to be mainly to enhance the regulation of the immune system, despite directly killing tumor cells. This mechanism may increase the lethality of CTX on tumor cells. Numerous reports indicate that elemene can improve the immune function of the body through many ways, and has protective and promoting effects on the immune system, such as induce cellular immune response, increase T cell transformation rate, activate macrophage activity, enhance tumor cell immunogenicity and attack tumors through immune molecules [39, 40]. (2) Free CTX directly binds to plasma proteins after entering the blood, and then releases into various tissues. However, the CTX in liposomes is slowly released and can be fully delivered to the tissues in the body, thereby reducing the clearance rate and improving the bioavailability. In other words, CTX encapsulated in liposomes can reduce the dosage to reach the plasma concentration of free CTX and thus reduce toxicity. (3) The strong permeability and the effect of inhibiting *P-gp* of ELE can overcome BBB and make CTX easier enter brain tumors to improve curative effect [41]. The findings of the current study imply that Tf-ELE/CTX@BLIP has good anti-glioma biocompatibility and is a promising nanoplatform for delivery of glioma chemotherapy.

## Conclusion

In summary, liposomes with active-targeting effects, excellent homologous recognition and BBB permeating capacities were prepared for combined treatment of glioma, providing a potential strategy for development of clinical chemotherapy of glioma. Tf-ELE/CTX@BLIP significantly improved transfer of ELE/CTX across the BBB and ingestion by glioma cells. MST of orthotopic glioma mice model was significantly longer and the liposomes improved biosafety of CTX, indicating an active-targeting biomimetic effectiveness. The findings provide a basis for further research for development of chemotherapy for central nervous system diseases. In addition, the findings provide ideas for development and research on active-targeting biomimetic nanoparticles.

## Material and methods

### Materials

ELE was purchased from Dalian Huali Jingang Pharmaceutical Co. Ltd (Dalian, China). CTX was purchased from Hubei Qianmo biological technology Co. Ltd (Hubei, China). Soya lecithin (the main ingredient is phosphatidylcholine, PC) was purchased from Shanghai Taiwei pharmaceutical Co. Ltd (Shanghai, China). d-α-tocopherol polyethylene glycol succinate (TPGS) was purchased from Wuhan Guobangda pharmaceutical chemical Co. Ltd (Wuhan, China). Medium chain triglyceride (MCT) was purchased from Xinxing Tieling pharmaceutical Co. Ltd (Tieling, China). Cholesterol (Chol) was purchased from Beijing Dingguo Changsheng Biotechnology Co. Ltd (Beijing, China). DSPE-PEG2000-transferrin was purchased from Xi’an Ruixi Biological Technology Co. Ltd (Xi’an, China). DAPI and GAPDH were purchased from Sigma-Aldrich (St Louis, USA). Penicillin–streptomycin and high-glucose DMEM, trypsin EDTA and FBS were purchased from Gibco Life Technologies (AG, USA). CCK-8 was purchased from Meilunbio (Dalian, China). Cypate was purchased from Hangzhou Xinqiao Biotechnology Co. LTD (Hangzhou, China). Coomassie blue, RIPA lysis buffer and bicinchoninic acid protein kit (BCA) were purchased from Biosharp (Shanghai, China). Apoptosis detection kit was purchased from Genview (USA).

### Cell culture

SPC-A-1 (human lung adenocarcinoma cell), A549 (lung cancer cell), MDA-MB-231 (human breast cancer cell), LM-3 (liver cancer cell), U251 glioma cells, C6 glioma cells, and RG2 glioma cells (Chinese Academy of Sciences, Shanghai, China) were cultured in high-glucose (4.5 g/L) DMEM supplemented with 10% FBS, 1% penicillin–streptomycin, 110 mg/L sodium pyruvate, and 2 mM l-glutamine and were incubated in normoxic conditions (5% CO_2_ and 37 °C).

### Isolation of CMP

CMP were extracted from RG2 glioma cells following a method reported previously with slight modifications [18, 42, 43]. RG2 cells were harvested and resuspended in 4 ℃ Tris-magnesium buffer (TM-buffer, pH 7.4) for 30 min, and crushed for 5 min using ultrasonic cell pulverizer (Xinyi-950E, 238 W, 2 s on, 3 s off). 1 mL 1 M sucrose solution was mixed with 3 mL of the cell debris and the homogenate was centrifuged to remove intracellular contents (2000×*g*, 10 min, 4 ℃). CMP were then obtained by further centrifugation (15,000×*g*, 30 min, 4 ℃) of the supernatant. Further, CMP were washed with pre-cooled TM-buffer (pH 7.4) containing 0.25 M sucrose, then centrifuged (15,000×*g*, 30 min, 4 ℃). CMP were then resuspended in PBS (1×) and attrition crushing was performed through 0.45 µm pores Protein quantity was determined by BCA protein assay for further preparation of liposomes. CMP from 3 × 10^8^ cells weighed 0.76 mg and were stored at – 20 ℃.

### Preparation and characterization of Tf-ELE/CTX@BLIP

Four types of liposomes including, ELE/CTX@LIP, Tf-ELE/CTX@LIP (ELE/CTX@LIP modified with Tf), ELE/CTX@BLIP (ELE/CTX@LIP modified with CMP), Tf-ELE/CTX@BLIP (ELE/CTX@LIP modified with both Tf and CMP) were constructed in the current study. The synthesis process of active-targeting biomimetic liposomes is presented in Fig. 1A ELE/CTX@LIP and Tf-ELE/CTX@LIP were prepared using high speed shear method combined with probe ultrasonic method [33, 40]. For preparation of Tf-ELE/CTX@LIP: 8 mg CTX was dissolved in 0.25 mL ethanol under a ultrasonic bath. 80 mg ELE, 20 mg cholesterol, 500 mg soybean lecithin, 100 mg TPGS, 10 mg DSPE-PEG2000-transferrin and 100 mg MCT were then added to the solution. The mixture was heated and dissolved under a 80 ℃ water bath to form the oil phase. In addition, 520 mg glycerol was dissolved in 18.5 g water, sheared at 3000 rpm at 60 ℃, to produce the aqueous phase. The oil phase was slowly injected into the aqueous phase, and shearing was performed at 60 ℃ and 10,000 rpm for 30 min (IKA^®^ T25 Easy Clean Digital Dispersion Machine, Germany). The particle size was then reduced using a ultrasonic sonicator for 10 min (Xinyi-950E ultrasonic cell pulverizer, China). ELE/CTX@LIP was prepared through the same process without addition of DSPE-PEG2000-transferrin. CMP-based liposomes were produced through a direct extrusion approach. Tf-ELE/CTX@BLIP was produced based on Tf-ELE/CTX@LIP, whereas ELE/CTX@BLIP was produced based on ELE/CTX@LIP. In summary, CMP were mixed with Tf-ELE/CTX@LIP or ELE/CTX@LIP at 100:1 phospholipid to CMP weight ratio [18]. The mixture was directly extruded through 0.45 µm and 0.22 µm pores to generate Tf-ELE/CTX@BLIP and ELE/CTX@BLIP.

Morphology of the four types of liposomes was explored using by transmission electron microscope (TEM, HITACHI HT7700 Exalens, Japan). Size distribution and ζ-potential of liposomes were determined by dynamic light scattering method (DLS, PSS Nicomp 380 Z3000 Zeta Potential and Nano particle Size meter, PSS, USA). Storage stability and serum stability of Tf-ELE/CTX@BLIP in water, PBS and PBS containing 10% FBS were explored by the diameter change over 7 days using DLS. CMP and Tf-ELE/CTX@BLIP protein profiles were determined by sodium dodecyl sulfate polyacrylamide gel electrophoresis (SDS-PAGE), and GAPDH as internal control then analyzed by western blot (WB). Encapsulation efficiency (EE) and drug loading efficiency (DLE) of ELE and CTX were determined by HPLC analysis (1260 Infinity II High Performance Liquid chromatograph, Agilent Technologies, USA). EE was then determine by the equation:

$${\text{EE}} = \left( {{\text{W}}_{{\text{a}}} /{\text{W}}_{{\text{a}}} + {\text{W}}_{{\text{b}}} } \right) \times {1}00\% {\text{ and DL}} = \left[ {{\text{W}}_{{\text{a}}} /{\text{W}}_{{\text{t}}} } \right)] \times {1}00\% ,$$

where W_a_ represents amount of ELE or CTX encapsulated in liposomes after hyperfiltration, Wb represents amount of ELE or CTX unencapsulated, and W_t_ represents the total amount of phospholipid.

Chromatographic conditions used for ELE included: Ultimata PAH chromatographic column (300 × 4.6 mm, 3 µm); mobile phase A: 75% acetonitrile-25% 0.01% phosphoric acid water; mobile phase B: 0.01% acetonitrile phosphate; elution conditions: 0–45 min, 80% A + 20% B, 45.01–65 min, 100% B; flow rate: 1.2 mL/min; injection volume: 20 µL; column temperature: 40 ℃ and the detection wavelength was 210 nm.

Chromatographic conditions used for CTX included: Agilent PFP chromatographic column (250 × 4.6 mm, 5 µm); mobile phase A: 51% acetonitrile–49% 0.01% phosphoric acid water; mobile phase B: 0.01% acetonitrile phosphate; elution conditions: 0–25 min, 100%, 25.01–45 min, 100% B; flow rate: 1.0 mL/min, injection volume: 20 µL, column temperature: 30 ℃ and the detection wavelength was 230 nm.

### In vitro cancer targeting study and cell uptake

Tf-ELE/CTX@BLIP was incubated with SPC-A-1 cells, A549 cells, MDA-MB-231 cells, LM-3 cells, U251 cells, C6 cells, and RG2 cells, separately. Cells were inoculated into 6-well plates (3 × 10^5^ cells/well) and incubated for 24 h. After incubation, the medium was replaced by fresh medium containing Tf-ELE/CTX@BLIP (Rhodamine B [Rho B] = 20 μg/mL) and incubated for 2 h. In addition, RG2 cells were seeded into a 6-well plate (3 × 10^5^ cells/well) and incubated with ELE/CTX@LIP, ELE/CTX@BLIP, Tf-ELE/CTX@LIP and Tf-ELE/CTX@BLIP (Rho B = 20 μg/mL) for 2 h. The medium was removed and cells were fixed with 4% paraformaldehyde, stained with DAPI and washed thrice with PBS. The cellular uptake was determined by confocal laser scanning microscope imaging (CLSM, FV3000RS, OLYMPUS, USA) and flow cytometry [44].

Macrophage RAW264.7 cells were exposed to ELE/CTX@LIP, ELE/CTX@BLIP, Tf-ELE/CTX@LIP and Tf-ELE/CTX@BLIP (Rho B = 20 μg/mL) for 2 h. Effect on immune evasion was then determined by CLSM and flow cytometry [45].

### In vitro cytotoxicity assay and cell apoptosis

Cytotoxicity effects of ELE/CTX@LIP, ELE/CTX@BLIP, Tf-ELE/CTX@LIP and Tf-ELE/CTX@BLIP on RG2 cells were determined using a CCK-8 kit. RG2 cells (3000 cells/well) were inoculated in 96-well plates and incubated for 24 h. After incubation, cells were exposed to ELE/CTX@LIP, ELE/CTX@BLIP, Tf-ELE/CTX@LIP and Tf-ELE/CTX@BLIP (CTX concentrations ranging from 0.4 to 200 ng/mL) for 48 h. Non-treated cells were used as negative controls. Medium was evaluated as the blank control. Cytotoxicity was quantitatively determined by measuring the absorbance at 450 nm using a Spark multi-functional microporous plate testing platform (Tecan, Switzerland).

Furthermore, apoptotic cells were determined by FACS analysis (CytoFLEX S, USA). RG2 cells were incubated with ELE/CTX@LIP, ELE/CTX@BLIP, Tf-ELE/CTX@LIP and Tf-ELE/CTX@BLIP and 50 ng/mL CTX for 48 h then treated with the apoptosis detection kit (Genview, USA) for 10 min. Percentage of apoptotic cells was determined using a FACS Calibur System. Non-treated cells were used as the negative control [46].

### Inhibitory effect of Tf-liposomes on *P-gp* function

bEnd.3 cells were cultured as described in the “Cell culture” section. Cells were inoculated into 6-well plates (5 × 10^5^ cells/well) and incubated for 24 h. Rhodamine 123 (*P-gp* substrate, 20 μg/mL) was preconditioned with ELE/CTX@LIP, ELE/CTX@BLIP, Tf-ELE/CTX@LIP, Tf-ELE/CTX@BLIP (50 ng/mL) and verapamil (0.625 μg/mL) separately for 30 min then it was exposed to bEnd.3 for 2 h. The medium was removed and bEnd.3 cells were fixed with 4% paraformaldehyde for 30 min and washed thrice with PBS. Cells were then analyzed using fluorescence microscope and flow cytometry. Relative expression level of *P-gp* in bEnd.3 cells was determined by WB assay. bEnd3 cells in 6-well plates were conditioned with medium and verapamil, ELE/CTX@LIP, ELE/CTX@BLIP, Tf-ELE/CTX@LIP, Tf-ELE/CTX@BLIP were added [47].

### Animal and orthotopic glioma-bearing model

Female nude mice (20 ± 2 g, BALB/c) were obtained from Shanghai Slack Laboratory Animal Co. LTD. Mice were allowed to acclimatize at room temperature for 7 days prior to the performing animal studies. Animals were housed under the animal care facility and allowed free access to food/water. All animal experiments were approved by the animal ethics committee of Hangzhou Normal University (HangZhou, China).

Glioma-luc cells were injected into the right striatum of nude mice to develop a orthotopic glioma-bearing model. Nude mice were anesthetized through administration of 10% chloral hydrate and the head was immobilized on a stoelting (Lab Standard, USA). Approximately 2.5 × 10^7^ glioma-luc cells in medium were injected slowly into the brain right striatum of nude mice (bright lateral: 2.0 mm, bregma: 1.8 mm, depth: 3.5 mm). The needle was maintained at the injection point for 1 min after injection and then pulled out slowly. Alcohol cotton swabs were used to disinfect the skin, the skin was sewn with a needle and thread. The mice were then placed back to the cage to wake up naturally. Rate of growth of intracranial tumor was monitored by magnetic resonance imaging (MRI) and fluorescence imaging.

### In vivo bioluminescence imaging assay

Orthotopic glioma-bearing model was established by injecting RG2 cells into the brain striatum as described above. Cypate, Cypate@LIP, Cypate@BLIP, Tf-Cypate@LIP and Tf-Cypate@BLIP (C_Cypate_ = 0.1 mg/mL) were injected into normal mice and glioma-bearing mice through the tail vein. Bioluminescence imaging was performed at fixed times (2, 8, 24 and 48 h) using a small animals in vivo 3D bioluminescence imaging system (IVIS Spectrum, PerkinElmer).The nude mice were then sacrificed, and brains, liver, heart, spleen, lung, and kidney were collected for quantitative biodistribution analysis and ex vivo bioluminescence imaging [48].

### In vivo anti-tumor study

Orthotopic glioma-bearing model nude mice were randomly assigned into seven groups (6 mice/group) as follows: (1) control (physiological saline); (2) CTX solution; (3) ELE solution (25 mg/kg ELE); (4) ELE/CTX@LIP; (5) ELE/CTX@BLIP; (6) Tf-ELE/CTX@LIP; (7) Tf-ELE/CTX@BLIP, to explore the anti-tumor effect in vivo. Treatments were administered by intravenous injection through the tail at day 1, 3, 5, 7, 9 and 11. Group 2, 4, 5, 6, 7 was administered with 2.5 mg/kg (CTX) on the first time and 0.625 mg/kg for the subsequent 5 injections. Bioluminescence imaging was performed at day 1, 5, 10, and 15 to explore tumor growth. Body weight and survival time were recorded every 3 days. Nude mice were euthanized 2 h post-treatment and their brains were collected for H&E staining, TUNEL immunofluorescence staining and detection of glioma *P-gp*.

### Toxicity evaluation

Glioma-bearing nude mice were assigned into seven groups and were administered with (1) control (physiological saline); (2) CTX solution; (3) ELE solution; (4) ELE/CTX@LIP; (5) ELE/CTX@BLIP; (6) Tf-ELE/CTX@LIP; (7) Tf-ELE/CTX@BLIP, 6 times and every other day. Whole blood samples were collected from retro-orbital sinus of glioma-bearing nude mice 2 h post-treatment. A portion of the complete blood samples was used for complete blood count analysis. The supernatant (serum) of the portion of blood obtained after centrifugation (5000 rpm, 10 min) was used for determination of biochemical indexes of liver and kidney, including total bilirubin, blood urea nitrogen, uric acid, alanine transaminase, creatinine and aspartate transaminase level. Liver, heart, spleen, lung and kidney sections were stained with H&E to evaluate effects of free ELE, CTX solution and liposomes toxicity [49].

### Statistical analysis

GraphPad Prism 8.0.2.263 software (GraphPad Software Inc., San Diego, CA, USA) was used for statistical analysis. Data were expressed as mean ± SD. Experimental data were analyzed using two-tailed Student *t* test for comparison between two groups and ANOVA for comparison among multiple groups. Statistical difference was defined as significant for *p < 0.05 and highly significant for **p < 0.01.

## Supplementary Information

**Additional file 1: Table S1.** Encapsulation efficiency stability of the drug in 4 liposomes at 3 months (n = 3). **Fig. S1.** Diameter and ζ-potential of Tf-ELE/CTX@BLIP, Tf-ELE/CTX@LIP, ELE/CTX@BLIP and ELE/CTX@LIP. **Fig. S2.** 7-days stability of TF-ELE/CTX@BLIP diameter in different medium. **Fig. S3.** Flow cytometry analysis of RG2 glioma cells after incubation with Tf-ELE/CTX@BLIP, Tf-ELE/CTX@LIP, ELE/CTX@BLIP and ELE/CTX@LIP for 2 h. Rho B = 20 μg/mL. **Fig. S4.** CLSM images of RG2, U251 and C6 glioma cells treated with Tf-ELE/CTX@BLIP for 2 h. Scale bar = 50 μm. **Fig. S5.** Flow cytometry analysis of RAW264.7 cells treated with Tf-ELE/CTX@BLIP, Tf-ELE/CTX@LIP, ELE/CTX@BLIP and ELE/CTX@LIP for 2 h. Rho B = 20 μg/mL. **Fig. S6.** WB analysis of P-gp in bEnd.3 cells. Relative protein expression was calculated. Cells preconditioned with (1) control; (2) verapamil; (3) ELE/CTX@LIP; (4) ELE/CTX@BLIP; (5) Tf-ELE/CTX@LIP; (6) Tf-ELE/CTX@BLIP. (*P < 0.05, n=3). **Fig. S7.** Corresponding quantitative fluorescent analysis of brain, liver, heart, spleen, lung and kidney at 48 h post-injection in glioma-beard mice. *p < 0.05. **Fig. S8.** In vivo fluorescence imaging of Tf-Cypate@BLIP, Tf-Cypate@LIP, Cypate@BLIP and Cypate@LIP in normal mice. Cypate = 0.5 mg/kg. **Fig. S9.** Averaged fluorescent intensity of saline, free CTX, free ELE, ELE/CTX@LIP, ELE/CTX@BLIP, Tf-ELE/CTX@LIP and Tf-ELE/CTX@BLIP in nude mice bearing orthotopic glioma brain within 15 days of treatments. **Fig. S10.** H&E staining of brain sections of orthotopic glioma-bearing mice in different formulation groups. **Fig. S11.** Biochemical parameter analysis after treated with saline, free CTX, free ELE, ELE/CTX@LIP, ELE/CTX@BLIP, Tf-ELE/CTX@LIP and Tf-ELE/CTX@BLIP. (A) BLI-T: total bilirubin, (B) BUN: blood urea nitrogen, (C) URIC: uric acid, (D) ALT: alanine transaminase, (E) CRE: creatinine, (F) AST: aspartate transaminase.

## Data Availability

All data generated or analyzed during this study are included in this article.

## Abbreviations

BBB: Blood–brain barrier
ELE: Elemene
CTX: Cabazitaxel
Tf: Transferrin
*P-gp*: P-glycoprotein
CMP: Cell membrane proteins
PC: Phosphatidylcholine
TPGS: d-α-tocopherol polyethylene glycol succinate
MCT: Medium chain triglyceride
Chol: Cholesterol
TEM: Transmission electron microscope
CLSM: Confocal laser scanning microscope imaging
MRI: Magnetic resonance imaging
DLS: Dynamic light scattering
EE: Encapsulation efficiency
DLE: Drug loading efficiency
WB: Western blot
SDS-PAGE: Sodium dodecyl sulfate polyacrylamide gel electrophoresis
MST: Median survival time
WBC: White blood cell
Lymph: Lymphocyte
Mon: Monocyte
Gran: Neutrophil granulocyte
P-Lymph: Percentage of lymphocyte
P-Mon: Percentage of monocyte
P-Gran: Percentage of neutrophil granulocyte
RBC: Red blood cell
HGB: Hemoglobin
HCT: Hematocrit
MCV: Mean corpuscular volume
MCH: Mean corpuscular hemoglobin
MCHC: Mean concentration of corpuscular hemoglobin
RDW: Red blood cell distribution width
PLT: Platelet
MPV: Mean platelet volume
