# Supplementary material for: Active targeting of orthotopic glioma using biomimetic liposomes co-loaded elemene and cabazitaxel modified by transferritin
Source: J Nanobiotechnology. 2021 Sep 26;19:289. doi: 10.1186/s12951-021-01048-3 (PMC8474941; doi:10.1186/s12951-021-01048-3)
Supplement: Supplementary file 1 — Additional file 1: Table S1. Encapsulation efficiency stability of the drug in 4 liposomes at 3 months (n = 3). Fig. S1. Diameter and ζ-potential of Tf-ELE/CTX@BLIP, Tf-ELE/CTX@LIP, ELE/CTX@BLIP and ELE/CTX@LIP. Fig. S2. 7-days stability of TF-ELE/CTX@BLIP diameter in different medium. Fig. S3. Flow cytometry analysis of RG2 glioma cells after incubation with Tf-ELE/CTX@BLIP, Tf-ELE/CTX@LIP, ELE/CTX@BLIP and ELE/CTX@LIP for 2 h. Rho B = 20 μg/mL. Fig. S4. CLSM images of RG2, U251 and C6 glioma cells treated with Tf-ELE/CTX@BLIP for 2 h. Scale bar = 50 μm. Fig. S5. Flow cytometry analysis of RAW264.7 cells treated with Tf-ELE/CTX@BLIP, Tf-ELE/CTX@LIP, ELE/CTX@BLIP and ELE/CTX@LIP for 2 h. Rho B = 20 μg/mL. Fig. S6. WB analysis of P-gp in bEnd.3 cells. Relative protein expression was calculated. Cells preconditioned with (1) control; (2) verapamil; (3) ELE/CTX@LIP; (4) ELE/CTX@BLIP; (5) Tf-ELE/CTX@LIP; (6) Tf-ELE/CTX@BLIP. (*P < 0.05, n=3). Fig. S7. Corresponding quantitative fluorescent analysis of brain, liver, heart, spleen, lung and kidney at 48 h post-injection in glioma-beard mice. *p < 0.05. Fig. S8. In vivo fluorescence imaging of Tf-Cypate@BLIP, Tf-Cypate@LIP, Cypate@BLIP and Cypate@LIP in normal mice. Cypate = 0.5 mg/kg. Fig. S9. Averaged fluorescent intensity of saline, free CTX, free ELE, ELE/CTX@LIP, ELE/CTX@BLIP, Tf-ELE/CTX@LIP and Tf-ELE/CTX@BLIP in nude mice bearing orthotopic glioma brain within 15 days of treatments. Fig. S10. H&E staining of brain sections of orthotopic glioma-bearing mice in different formulation groups. Fig. S11. Biochemical parameter analysis after treated with saline, free CTX, free ELE, ELE/CTX@LIP, ELE/CTX@BLIP, Tf-ELE/CTX@LIP and Tf-ELE/CTX@BLIP. (A) BLI-T: total bilirubin, (B) BUN: blood urea nitrogen, (C) URIC: uric acid, (D) ALT: alanine transaminase, (E) CRE: creatinine, (F) AST: aspartate transaminase. [file 12951_2021_1048_MOESM1_ESM.docx]

**Table S1** Encapsulation efficiency stability of the drug in 4 liposomes at 3 months (n = 3).

| **Samples** | **Size/nm** | **PDI** | **ζ- poteneial/mV** | **EE (%)-after 3 months of storage** | | |
| --- | --- | --- | --- | --- | --- | --- |
|  |  |  |  | **ELE** | | **CTX** |
| Tf-ELE/CTX@BLIP | 135.1±4.2 | 0.263±0.018 | 33.57±0.67 | 92.580±0.068 | 96.883±0.113 | |
| Tf-ELE/CTX@LIP | 153.0±3.4 | 0.345±0.011 | 54.96±1.91 | 93.512±0.117 | 97.860±0.361 | |
| ELE/CTX@BLIP | 115.7±1.3 | 0.168±0.023 | 33.95±1.08 | 92.327±0.295 | 97.698±0.662 | |
| ELE/CTX@LIP | 135.4±2.0 | 0.205±0.021 | 34.53±2.66 | 93.818±0.516 | 94.197±0.378 | |
| CMP | - | - | 30.12±0.73 | - | - | |


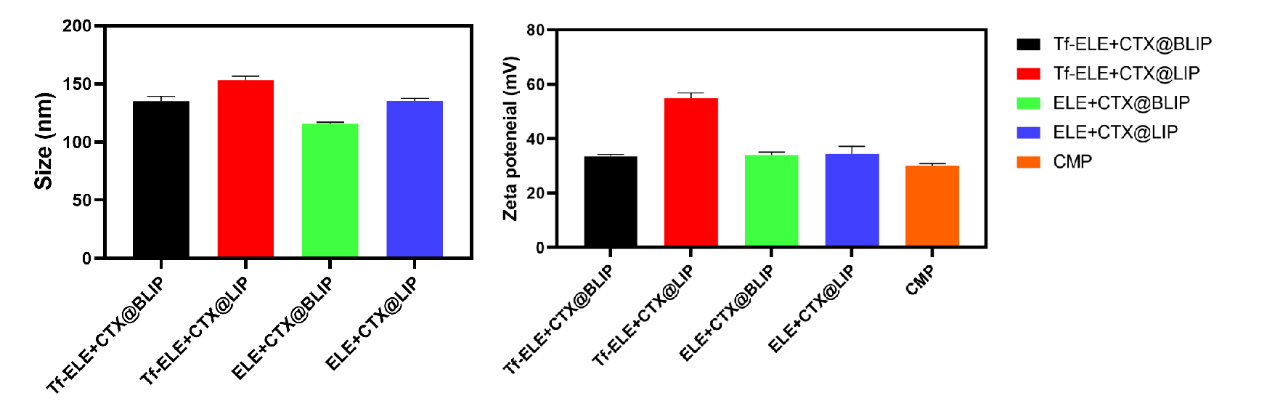


**Fig. S1** Diameter and ζ-poteneial of Tf-ELE/CTX@BLIP, Tf-ELE/CTX@LIP,

ELE/CTX@BLIP and ELE/CTX@LIP.


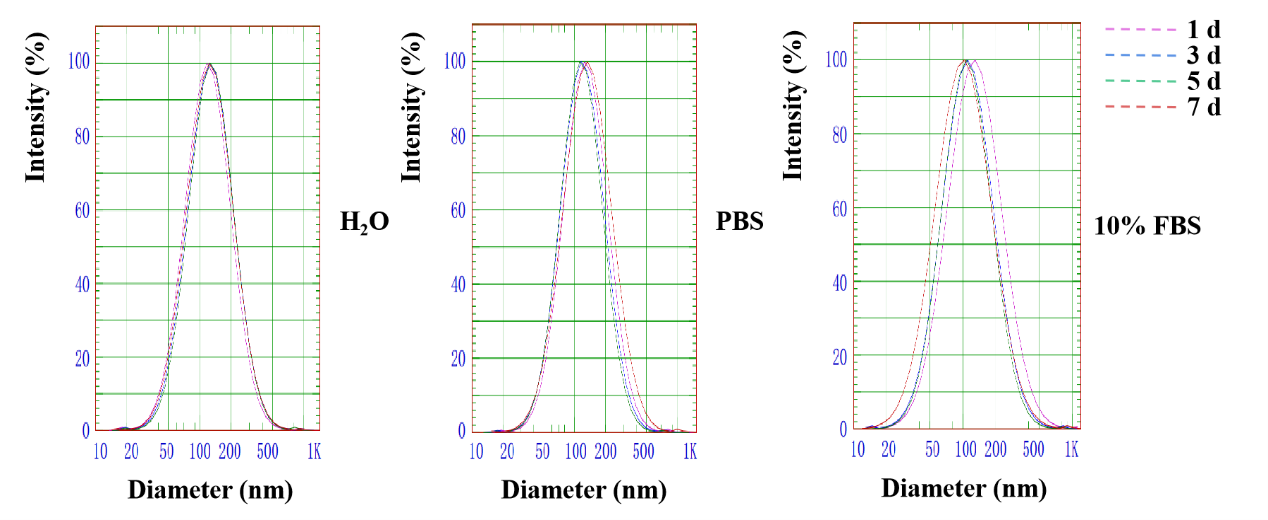


**Fig. S2** 7-days stability of TF-ELE /CTX@BLIP diameter in different medium.


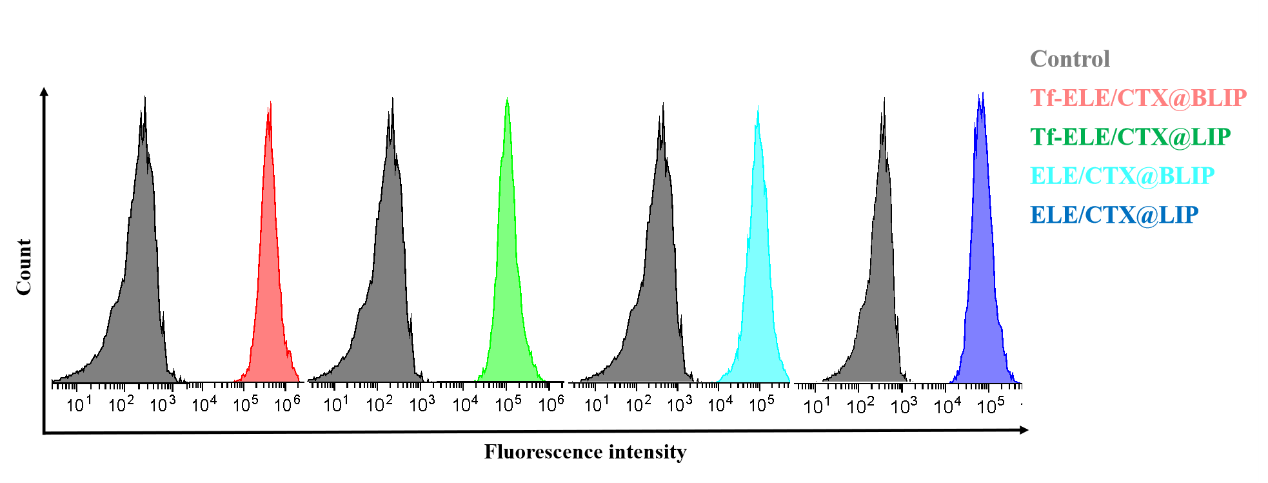


**Fig. S3** Flow cytometry analysis of RG2 glioma cells after incubation with Tf-ELE/CTX@BLIP, Tf-ELE/CTX@LIP, ELE/CTX@BLIP and ELE/CTX@LIP for 2 h. Rho B = 20 μg/ mL.

**
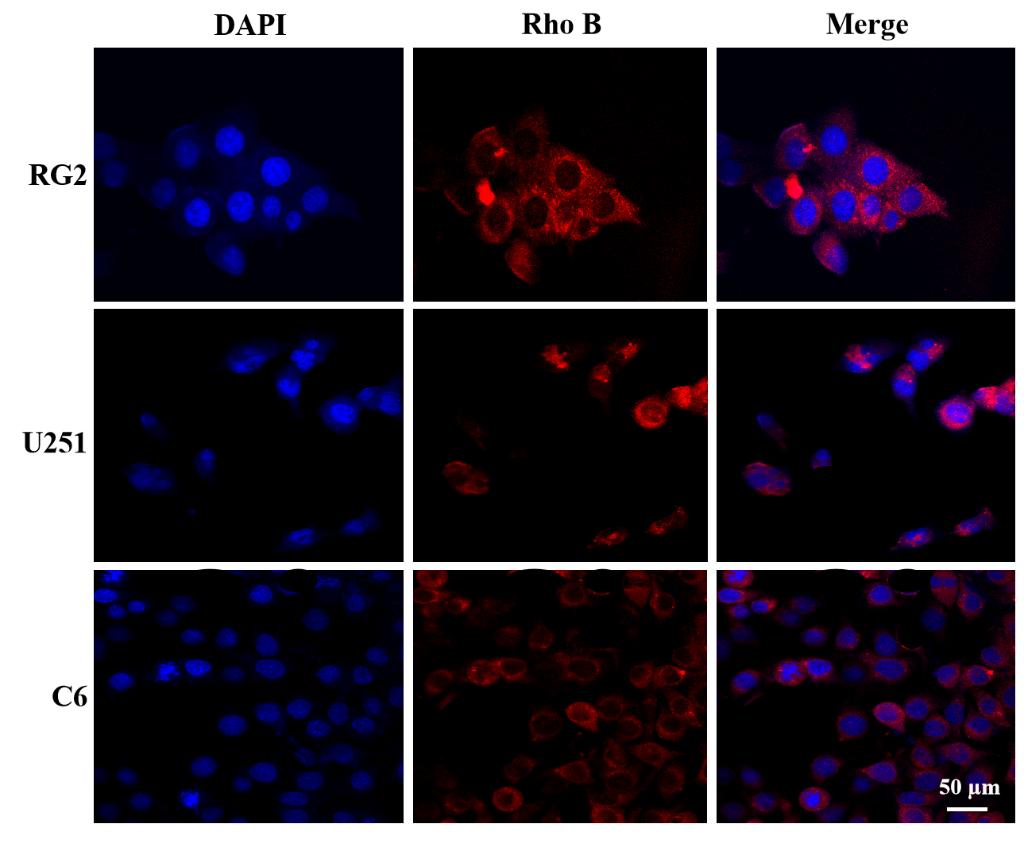
**

**Fig. S4** CLSM images of RG2, U251 and C6 glioma cells treated with Tf-ELE/CTX@BLIP for 2 h. Scale bar = 50 μm.

**
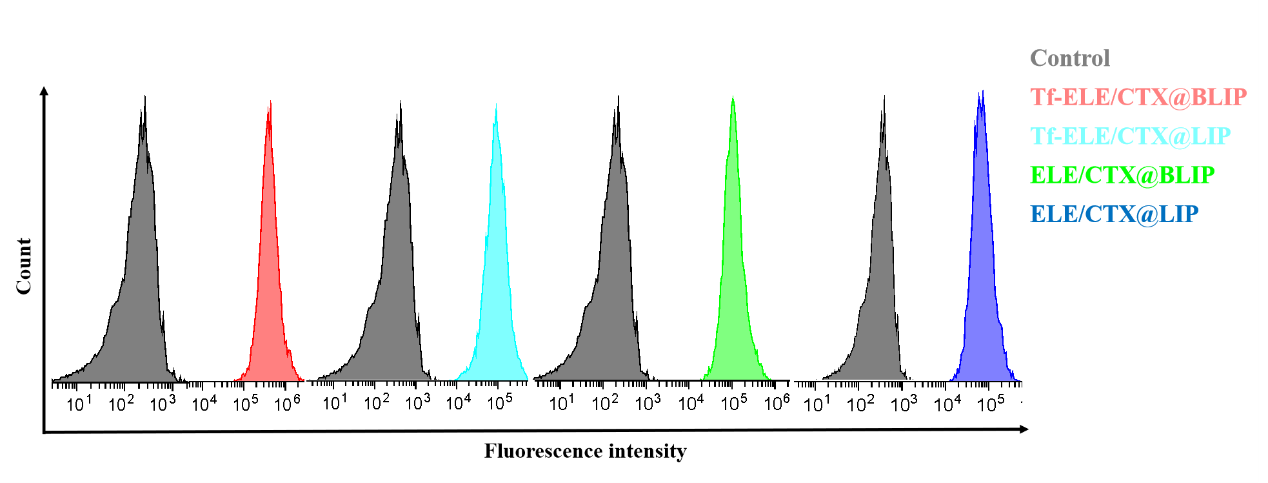
**

**Fig. S5** Flow cytometry analysis of RAW264.7 cells treated with Tf-ELE/CTX@BLIP, Tf-ELE/CTX@LIP, ELE/CTX@BLIP and ELE/CTX@LIP for 2 h. Rho B = 20 μg/ mL.

**
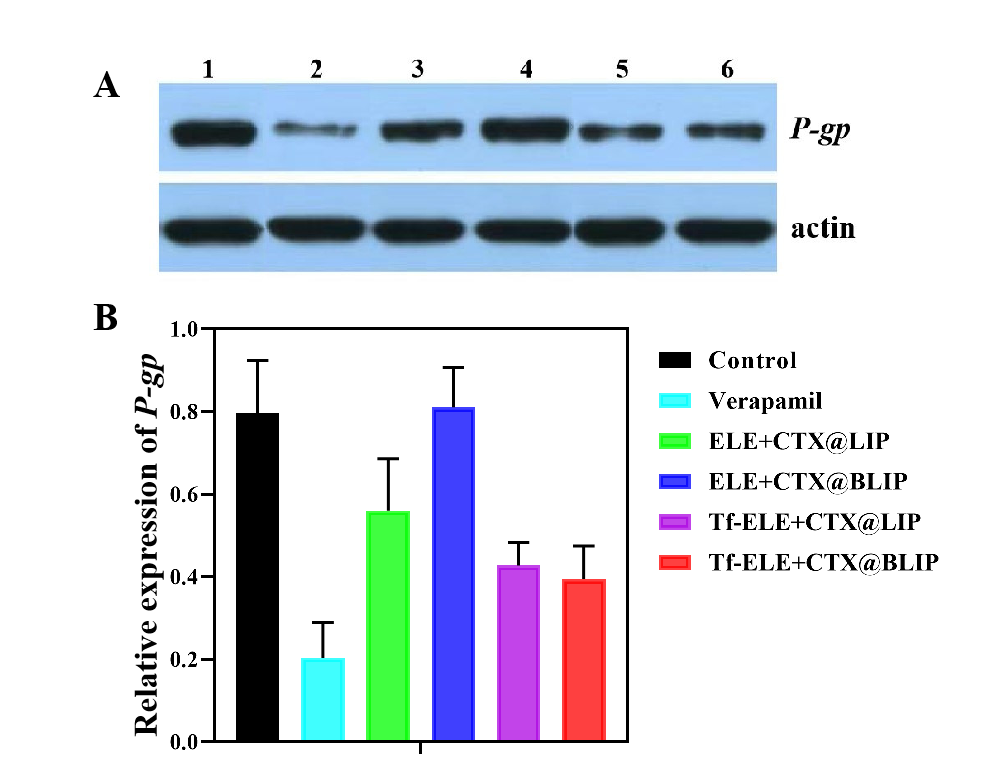
**

**Fig. S6** WB analysis of *P-gp* in bEnd.3 cells. Relative protein expression was calculated. Cells preconditioned with (1) control; (2) verapamil; (3) ELE/CTX@LIP; (4) ELE/CTX@BLIP; (5) Tf-ELE/CTX@LIP; (6) Tf-ELE/CTX@BLIP. (**P* < 0.05, n=3).

**
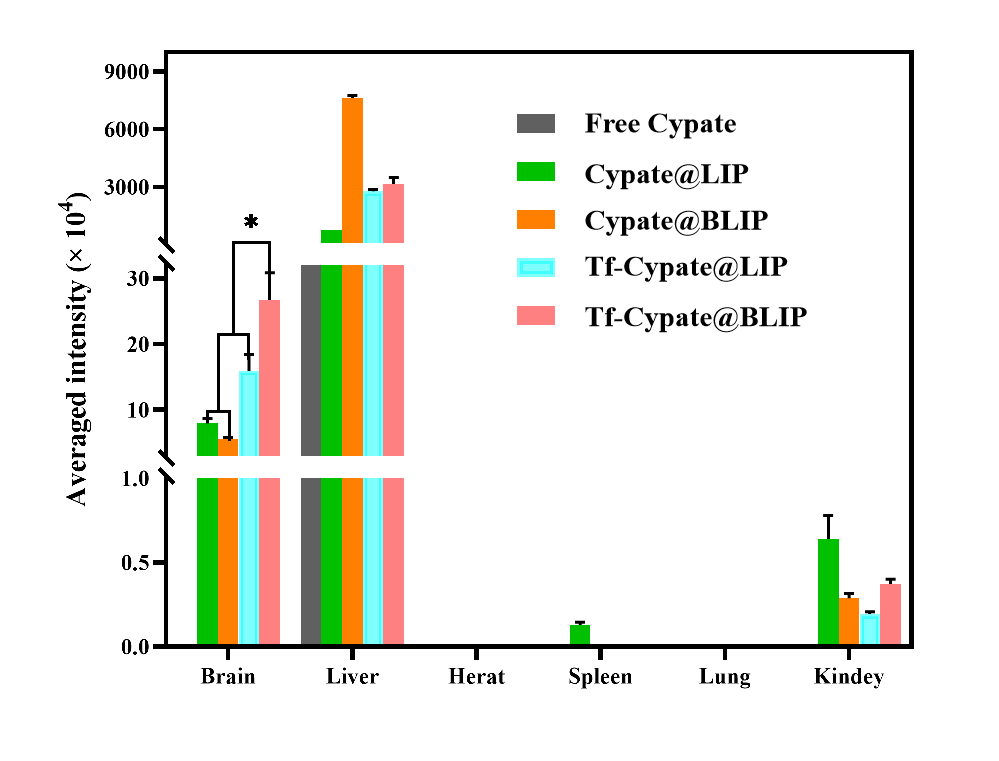
**

**Fig. S7** Corresponding quantitative fluorescent analysis of brain, liver, heart, spleen, lung and kidney at 48 h post-injection in glioma-beard mice. **p* < 0.05.


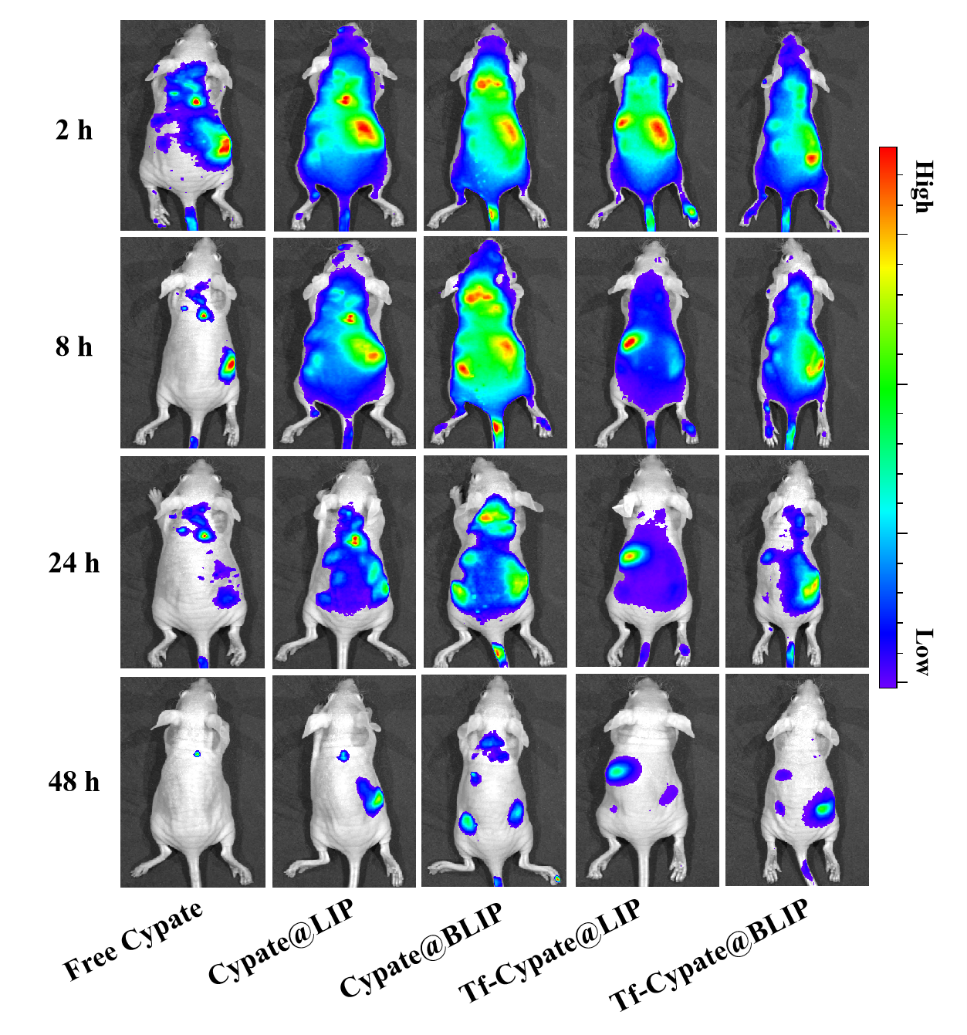


**Fig. S8** *In vivo* fluorescence imaging of Tf-Cypate@BLIP, Tf-Cypate@LIP, Cypate@BLIP and Cypate@LIP in normal mice. Cypate= 0.5 mg/kg.

*
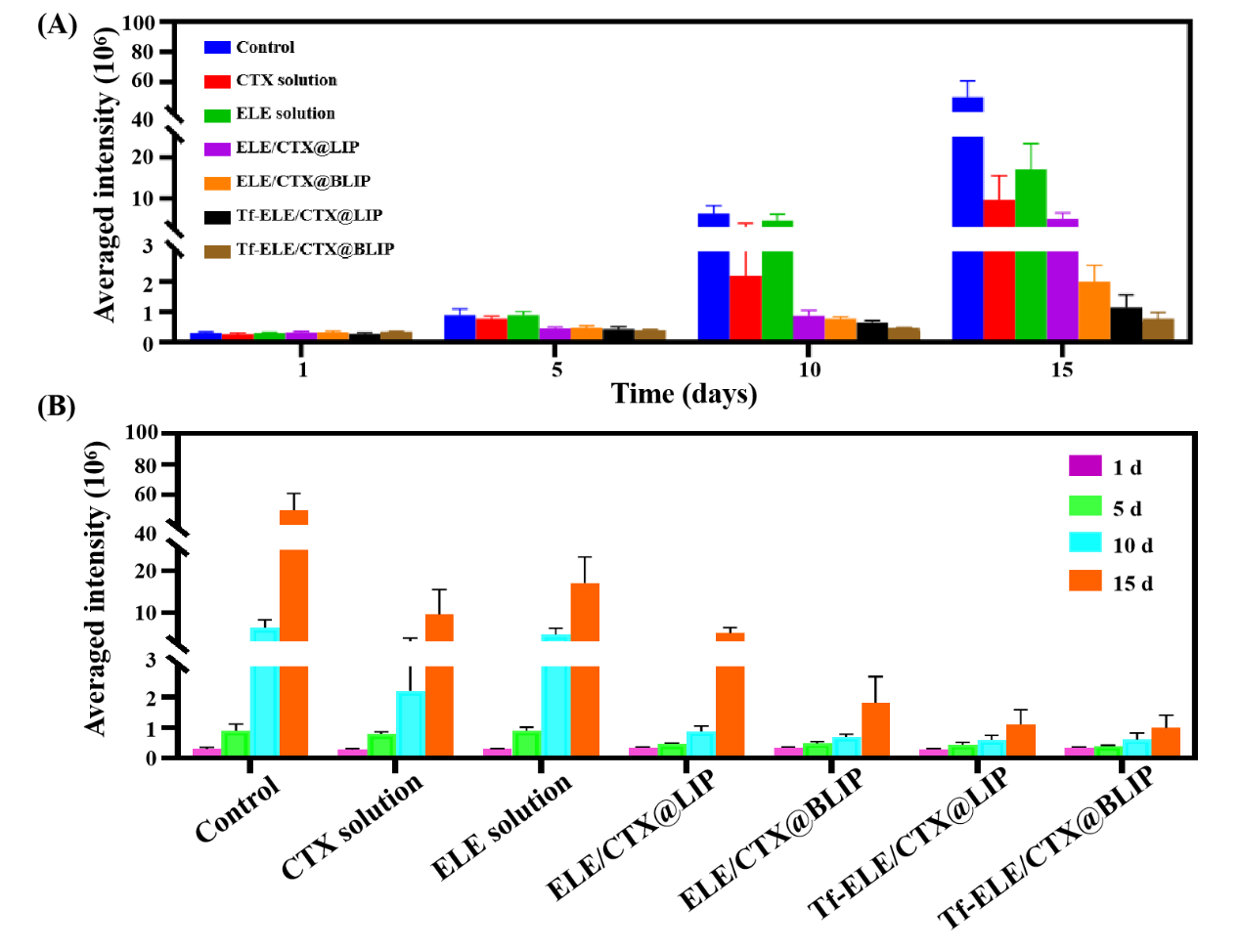
*

**Fig. S9** Averaged fluorescent intensity of saline, free CTX, free ELE, ELE/CTX@LIP, ELE/CTX@BLIP, Tf-ELE/CTX@LIP and Tf-ELE/CTX@BLIP in nude mice bearing orthotopic glioma brain within 15 days of treatments.

**
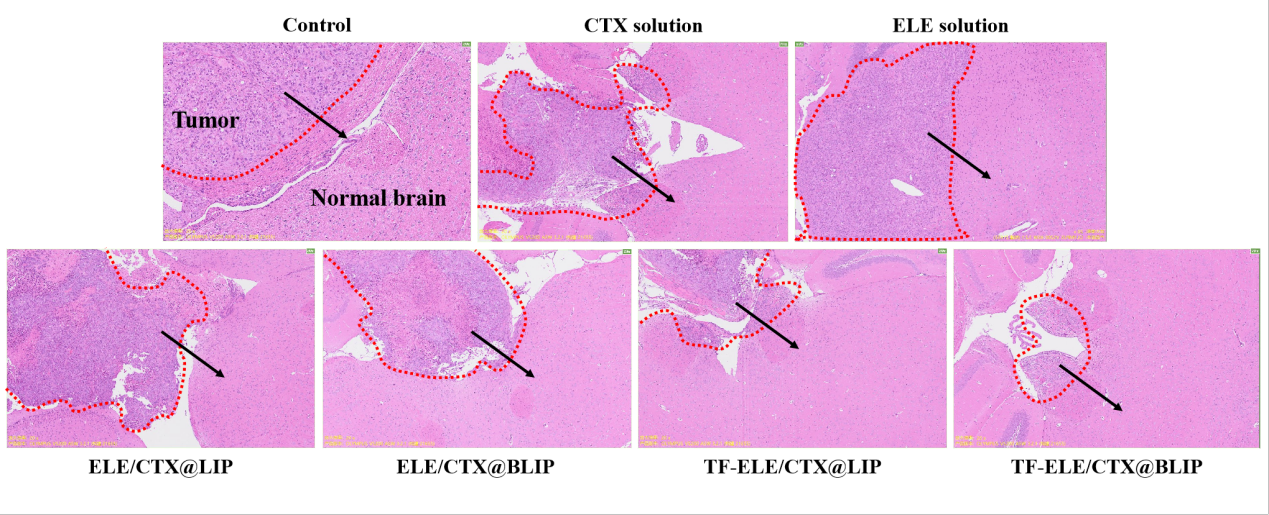
**

**Fig. S10** H&E staining of brain sections of orthotopic glioma-bearing mice in different formulation groups.


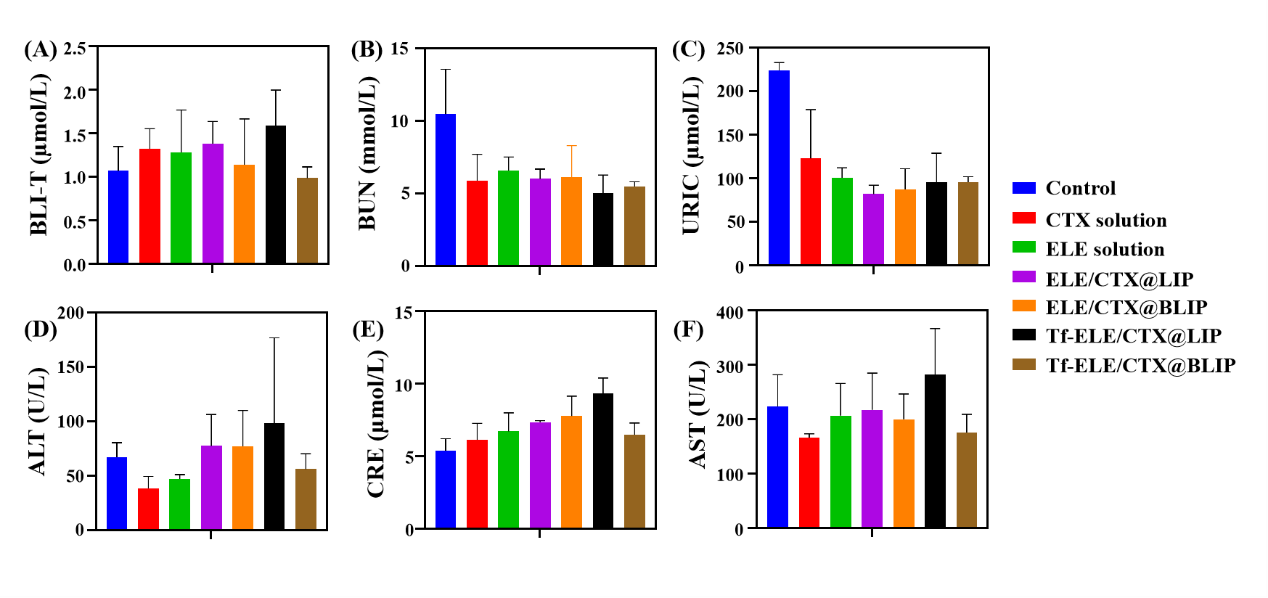


**Fig. S11** Biochemical parameter analysis after treated with saline, free CTX, free ELE, ELE/CTX@LIP, ELE/CTX@BLIP, Tf-ELE/CTX@LIP and Tf-ELE/CTX@BLIP. (A) BLI-T: total bilirubin, (B) BUN: blood urea nitrogen, (C) URIC: uric acid, (D) ALT: alanine transaminase, (E) CRE: creatinine, (F) AST: aspartate transaminase.
